# The Role of Natural Products in Drug Discovery and Development against Neglected Tropical Diseases

**DOI:** 10.3390/molecules22010058

**Published:** 2016-12-31

**Authors:** Peter Mubanga Cheuka, Godfrey Mayoka, Peggoty Mutai, Kelly Chibale

**Affiliations:** 1 Department of Chemistry, University of Cape Town, Rondebosch 7701, South Africa; chkpet008@myuct.ac.za (P.M.C.); mykgod001@myuct.ac.za (G.M.); 2 Department of Pharmacology and Pharmacognosy, University of Nairobi, P.O. Box 19676, 00202 KNH Nairobi, Kenya; pckemei@gmail.com; 3 Institute of Infectious Disease and Molecular Medicine, University of Cape Town, Rondebosch 7701, South Africa; 4 South African Medical Research Council Drug Discovery and Development Research Unit, University of Cape Town, Rondebosch 7701, South Africa

**Keywords:** human African trypanosomiasis, leishmaniasis, schistosomiasis, lymphatic filariasis, natural products, antiprotozoal agents, phytotherapy

## Abstract

Endemic in 149 tropical and subtropical countries, neglected tropical diseases (NTDs) affect more than 1 billion people annually, including 875 million children in developing economies. These diseases are also responsible for over 500,000 deaths per year and are characterized by long-term disability and severe pain. The impact of the combined NTDs closely rivals that of malaria and tuberculosis. Current treatment options are associated with various limitations including widespread drug resistance, severe adverse effects, lengthy treatment duration, unfavorable toxicity profiles, and complicated drug administration procedures. Natural products have been a valuable source of drug regimens that form the cornerstone of modern pharmaceutical care. In this review, we highlight the potential that remains untapped in natural products as drug leads for NTDs. We cover natural products from plant, marine, and microbial sources including natural-product-inspired semi-synthetic derivatives which have been evaluated against the various causative agents of NTDs. Our coverage is limited to four major NTDs which include human African trypanosomiasis (sleeping sickness), leishmaniasis, schistosomiasis and lymphatic filariasis.

## 1. Introduction

### 1.1. Neglected Tropical Diseases: Definition, Classification and Epidemiology

According to the World Health Organization (WHO), 17 diseases caused by bacteria and parasites, are classified as neglected tropical diseases (NTDs) [1]. These include Buruli ulcer, Chagas disease, dengue, chikungunya, dracunculiasis (Guinea-worm disease), echinococcosis, foodborne trematodiases, human African trypanosomiasis (sleeping sickness), and leishmaniasis. More recently, mycetoma was also officially recognized as a NTD at the 69th World Health Assembly held on 28 May 2016. Because there are still other diseases in poverty-stricken settings that remain neglected, the World Health Assembly also resolved to systematically evaluate and potentially include more diseases to the list of NTDs. Populations living in poverty with sub-standard sanitation and close contact with infectious vectors as well as domestic animals and livestock are the most affected. A general lack of attention to the developing world can explain why these diseases have been neglected for decades. More recently, diseases like HIV/AIDS, tuberculosis, and malaria have drawn much of the attention and research funding at the expense of NTDs [2].

The WHO-recognized NTDs can further be categorized according to the class of the disease-causing agents. For instance, those caused by protozoan parasites include human African trypanosomiasis, otherwise called sleeping sickness. This is caused by two parasites of the genus *Trypanosoma*—*T. brucei gambiense* and *T. brucei rhodesiense* [2]. *Trypanosoma cruzi* represents another protozoan parasite that is responsible for Chagas disease, an infection that affects about 200,000 new people per year in the Americas and is one of the human parasitic conditions that is deeply concerning to the New World [3]. Furthermore, leishmaniasis is also caused by a myriad of species of protozoan parasites of the genus *Leishmania*. These parasites have been implicated in a range of disease conditions which can be self-healing ulcers on the one hand while severe and fatal visceral disease can also occur. With the exception of malaria and lymphatic filariasis, this group of parasites has been associated with the highest mortality and morbidity among human parasitic infections [4]. Another major class of NTDs is caused by helminths which include soil-transmitted helminths (STHs), schistosomiasis, lymphatic filariasis, onchocerciasis, and dracunculiasis. At least a billion people are infected by one or more of the parasitic STHs with infection being facilitated by the ingestion of, as well as direct contact with, soil contaminated with worm eggs or larvae [5]. This group of parasitic infections are more devastating in children with a range of symptoms including stunted growth, compromised physical fitness, educational performance and school attendance [6]. This group of infections has significant poverty-inducing impact albeit death is an unlikely outcome [7]. Schistosomiasis, also known as bilharzia, represents another NTD caused by helminths with about 200 million people infected globally [8]. Humans are known to be infected by five species of parasitic trematodes belonging to the family Schistosomatidae. With snails known to be intermediate hosts, their life cycle is complicated. The parasites released into bodies of water by snails invade the human host through entry via intact human skin where they undergo further development in the lungs before migration to other tissues of the human body. Fever, lethargy and eosinophilia are some of the symptoms that characterize acute illness, also known as, Katayama fever. Tissue-specific pathologies depend on the species of schistosoma responsible for infection whereby *S. mansoni* and *S. japonicum* cause intestinal manifestations whereas *S. haematobium* leads to urinary schistosomiasis typified by haematuria-the presence of blood in urine [2]. Another psychologically and socio-economically devastating helminth infection is lymphatic filariasis, commonly known as elephantiasis. It is associated with enlargement of the limbs, genitals or breasts [9]. Endemic areas harbour 20% of the global population with over 120 million people in 83 countries infected. Of these, 40 million are disfigured by the disease [2]. Transmission is mediated by mosquitoes with most infections acquired remaining asymptomatic for many years. Other helminth-caused NTDs include onchocerciasis (river blindness) and dracunculiasis whose manifestation, symptoms as well as epidemiology are described elsewhere [2]. Another class of NTDs which include trachoma, Buruli ulcer and leprosy are caused by bacteria. Trachoma remains a public health concern in 42 countries and inflicts blindness or visual impairment of about 1.9 million people [10]. Contact with eye and nose discharges from infected individuals transmits the disease-causing bacterium *Chlamydia trachomatis*. Inanimate objects such as towels and/or washcloths as well as eye-seeking flies can also facilitate transmission [10]. Latest estimates indicate 200 million people are resident in endemic districts and are thus at risk of trachoma blindness [10].

### 1.2. Relative Public Health Impact

Endemic in 149 tropical and subtropical countries, NTDs affect more than 1 billion people including 875 million children, financially draining developing economies annually [1,11]. NTDs are responsible for over 500,000 deaths per year and are characterized by long-term disability and severe pain. Children suffer the most devastating symptoms which include malnutrition, cognitive impairment, stunted growth, and inability to attend school while infection in adults can result in social isolation as well as inability to work [11]. The impact of the combined NTDs closely rivals that of malaria and tuberculosis [12]. Some scholars have also argued that the reported health impact of NTDs may be underestimated due to the fact that many NTD infections are asymptomatic and are associated with longer incubation periods. In this regard, it has been argued that it is often difficult to establish a connection between a particular death and the corresponding NTD infection that has been latent over a long time [13].

### 1.3. Challenges in Drug Discovery for NTDs and Challenges/Limitations of Current Therapies

Drug discovery and development for NTDs is faced with a number of challenges. Firstly, investment in these therapeutic areas by major pharmaceutical companies is not financially attractive owing to the prospect of poor financial returns. For this reason, drug discovery against parasitic diseases, including NTDs, has not been motivated by commercial reasons [14]. Many pharmaceutical companies have adopted an opportunistic approach by utilizing drugs which were historically developed for other disease indications for repurposing against NTDs. Although such an approach has obvious advantages, which include the reduced cost of development, it does not, unfortunately, introduce chemically novel drugs on the market. Furthermore, the utility of such an approach may not be viable any more due to widespread resistance to certain chemical classes [14]. Since NTDs affect resource-constrained countries, it is usually a challenge to tailor drug candidates’ target product profiles to what is required in resource-poor settings. For instance, optimising a drug candidate for safe use without close medical supervision is one such obstacle [14].

Current clinically used drugs against NTDs are far from ideal. Some of the limitations associated with current chemotherapeutic agents include widespread drug resistance, severe adverse effects, lengthy treatment duration, unfavourable toxicity profiles, and complicated drug administration procedures—which may be a challenge in the resource-poor communities affected by the NTDs. The use of some drug regimens is also jeopardized by their limited availability [14].

### 1.4. Natural Products and Their Utility as Therapeutics: Advantages and Disadvantages

It has been estimated that approximately over half of the pharmaceuticals in clinical use today are derived from natural products [15]. Some natural product-derived drugs that are a hallmark of modern pharmaceutical care include quinine, theophylline, penicillin G, morphine, paclitaxel, digoxin, vincristine, doxorubicin, cyclosporine and vitamin A among many other examples [15]. For centuries, natural substances, particularly plants, have been used to control and treat diseases and this has culminated in the discovery of the majority of modern pharmaceutical agents [16]. Ancient Egyptians practiced medicine from as far back as 2900 BC. The “Ebers Papyrus”, the best known first record of Egyptian pharmaceutical practice has been dated to as far back as 1500 BC. The papyrus, which describes over 700 drugs, mostly of plant origin, details different drug formulations including gargles, snuffs, poultices, infusions, pills and ointments, with beer, milk, wine and honey being commonly used as vehicles [17]. Dioscorides first documented the medicinal use of natural substances as far back as 78 AD. Until now, thousands of medicinal plants described then remain relevant today in modern medicine [18]. Although such plant materials are no longer used as crude drug preparations, they remain important sources of purified active principles that have become cornerstones of modern therapy [15]. Between 2005 and 2007 alone, 13 natural-product-derived drugs were approved [19]. Other recently approved drugs, covered extensively in specialised reviews [17,19,20,21], include compounds that are derived from plant, microbial, and animal sources as well as semi-synthetic compounds based on natural product templates. Apart from covering a wide spectrum of disease indications—anti-cancer, anti-infective, anti-diabetic among others, they also show an exceptional diversity of chemical structures. Analysis of the chemical properties of recently developed small molecule natural-product-derived drugs has revealed that half of them were in conformity with Lipinski’s Rule of 5 for orally available drugs [22]. Although the remainder of the drugs had higher molecular weights, more rotatable bonds, and more stereogenic centres, they still exhibited relatively low log *p* values. Natural products, therefore, generally are more readily absorbed than synthetic drugs.

Despite such advantages and a proven track record of successes, many big pharmaceutical companies have been discouraged from pursuing natural product-based drug discovery due to perceived disadvantages of natural products. These include challenges in ensuring access and adequate supply, the complexities of natural product chemistry with associated slowness of working with natural products, and intellectual property rights concerns. Furthermore, the use of combinatorial chemistry to generate large libraries of synthetic compounds gave hope to pharmaceutical companies much to the detriment of natural products-based drug discovery research [23,24,25]. In this article, we review different natural products from plant, microbial, and marine sources that have shown potential as lead compounds against the neglected tropical diseases. The coverage has been largely restricted to only those natural products which have exhibited substantial potency against the different disease-causing agents. Other natural products evaluated against different NTD-causative agents have been extensively reviewed elsewhere [26,27,28,29,30,31,32,33,34,35,36,37,38,39,40,41,42,43,44,45].

## 2. Human African Trypanosomiasis (Sleeping Sickness)

### 2.1. Background of the Disease

As noted earlier in this review, the protozoan parasites of the genus *Trypanosoma* are responsible for the NTD human African trypanosomiasis. Commonly called sleeping sickness, the disease is transmitted by vectors—the bites of tsetse flies (*Glossina* spp.) facilitate entry of the parasites into the human host [46]. The parasites further invade the central nervous system upon multiplication and subsequent crossing of the blood-brain barrier. It is at this stage that the more obvious symptoms of the disease are evident: changes of behaviour, confusion, sensory disturbances and poor coordination. Another important symptom which gives the disease its name is the disruption of the sleep cycle with victims usually uncontrollably sleeping during the day [47]. The disease is usually fatal if not promptly diagnosed and treated. Of the two parasite species responsible for the disease, *T. brucei gambiense* accounts for 98% of reported cases. Due to sustained control efforts, the number of reported cases has significantly dropped from about 10,000 in 2009 to 3796 cases in 2014 [47].

The disease burden differs from one country to another with variations in different localities of the same country [47]. The Democratic Republic of the Congo (DRC) accounted for over 70% of the reported cases in the last decade. Additionally, 85% (over 1000 new cases) of the reported cases in 2014 were in the DRC. In the same year, between 100 and 200 cases were declared in the Central African Republic making it the second most afflicted country after the DRC. Comfortingly, other countries (Angola, Burkina Faso, Cameroon, Chad, Congo, Côte d’Ivoire, Gabon, Guinea, Malawi, South Sudan, Uganda, United Republic of Tanzania, Zambia and Zimbabwe) are reporting less than 100 cases annually [47]. Other African countries have not reported any new cases for over a decade. Transmission may have stopped in some of these countries albeit it remains a challenge to assess the exact situation in some areas beset by social and political instability [47].

### 2.2. Antitrypanosomal Drugs and Current Shortfalls

**Figure 1:**
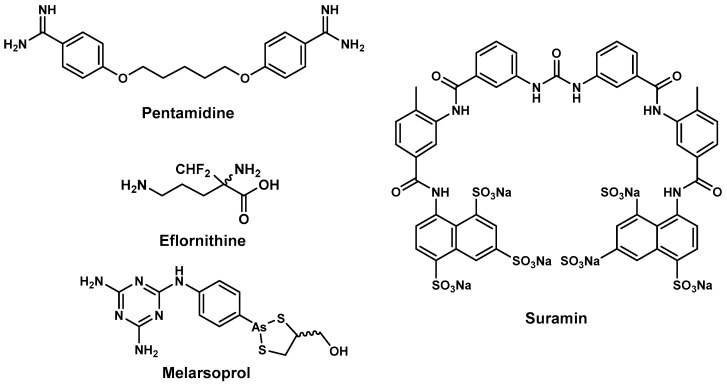
Antitrypanosomal drugs in clinical use.

Current antitrypanosomal drugs such as pentamidine, suramin, melarsoprol, and eflornithine, (Figure 1) are beset by a number of challenges ranging from limited effectiveness, emergence of drug resistance, complexity of administration, and too many undesirable side effects [47,48]. The disease causative pathogen and the stage of disease progression are the two factors that are considered when selecting drug treatment regimens. Currently, all the drugs are donated to the WHO by pharmaceutical companies [49,50]. For first-stage *T. brucei gambiense* disease, pentamidine is the drug of choice for treatment. Administration is complicated, often requiring intravenous infusion in saline over 2 h or intramuscular administration for a week. Although generally well tolerated, when administered via intramuscular injection, adverse events including pain and swelling at the site of administration, abdominal pain, gastrointestinal problems, and hypoglycaemia (5%–40%) have been reported [51].

First stage infections with *T. brucei rhodesiense* disease are generally treated with suramin, whose dose regimen is complicated and lasts up to 30 days [52]. Owing to its instability in air, the drug should be administered immediately upon dilution with distilled water [53]. Treatment with suramin is also accompanied with adverse side effects which include nephrotoxicity, peripheral neuropathy, bone marrow toxicity coupled with agranulocytosis and thrombocytopenia [54].

Second-line drugs that are used for treatment of the second phase of disease are melarsoprol and eflornithine. For treatment of second-phase disease caused by *T. brucei gambiense* in resource-constrained countries where availability/affordability of eflornithine is a challenge, the arsenic-containing compound, melarsoprol, is the drug of choice. It is also the only treatment option for second phase *T. brucei rhodesiense* infections. Treatment schedules with melarsoprol are complicated and are accompanied by severe adverse reactions which can be fatal [51]. Developed nearly three decades ago, eflornithine offers a safer treatment option [55] for *T. brucei gambiense* disease although side effects including fever, unusual bleeding and weakness, diarrhoea, nausea, stomach pain, as well as vomiting are common [56]. In the context of rural Africa, the use of eflornithine is severely limited by the need to administer it as multiple daily infusions [57].

### 2.3. Antitrypanosomal Natural Products

Natural products represent an untapped resource for novel, diverse, safe, and affordable anti-trypanosomal drug lead compounds. Nature has inspired a number of human antiprotozoal chemotherapeutics, which include antimalarials such as quinine, an alkaloid from *Cinchona* spp. (Rubiaceae), and artemisinin, a sesquiterpene lactone derived from *Artemisia annua* (Asteraceae). Used to treat amoebiasis, emetine is another antiprotozoal alkaloid from *Cephaelis ipecacuanha* (Rubiaceae). Additionally, these naturally-derived antiprotozoal agents have been employed as templates in the development of synthetic and semisynthetic drugs with improved efficacy, safety or pharmacokinetic properties [58]. An extensive literature search revealed several natural-product-based classes of antitrypanosomal molecules albeit very few have been advanced to in vivo evaluation while none have been clinically studied [32]. As we review the different antitrypanosomal natural product molecules, an attempt has been made to classify them based on chemical class. Natural products from plant, microbial and marine sources are reviewed. Total synthetic and semisynthetic compounds based on natural product scaffolds are also reviewed. All IC_50_ values reported in this review have been harmonized by converting to micromolar (μΜ) values. There is also need to exercise caution in drawing comparisons between IC_50_ values for different metabolites. This is because of variations in the nature of assays used in experimentally determining such values in different research laboratories. The review is restricted in scope, emphasising only those natural products that have exhibited significant potency in vitro (IC_50_ < 1 μΜ) and in vivo against trypanosomes responsible for human African trypanosomiasis. A similar restriction was also applied for antileishmanial natural products, which are discussed in later sections of this review.

#### 2.3.1. Antitrypanosomal Natural Products from Marine Sources

##### Alkaloids

**Figure 2:**
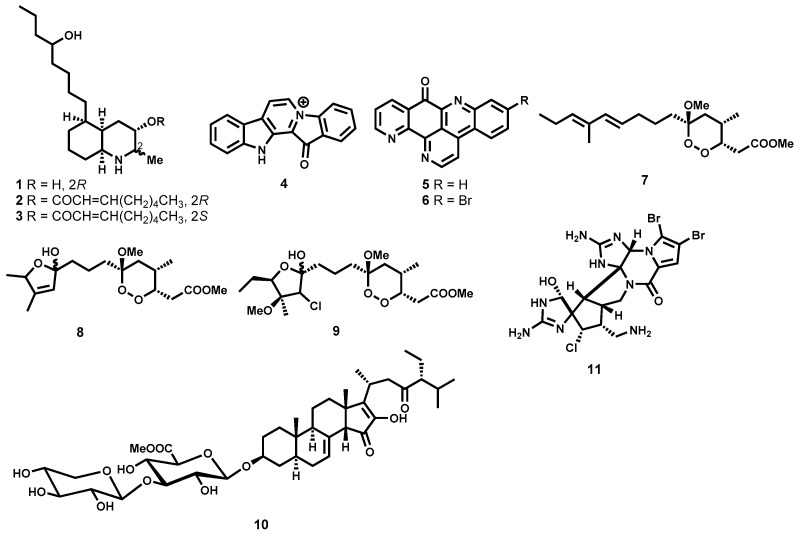
Trypanocidal alkaloids, saponins, and peroxides isolated from marine organisms.

Antitrypanosomal activity of several alkaloids isolated from marine organisms which include sponges, ascidians and tunicates has been reported [59,60,61]. In this regard, lepadins D (**1**), E (**2**), and F (**3**) (Figure 2), isolated from the tunicate species of the genus *Didemnum*, and possessing a decahydroquinoline skeleton, demonstrated significant antitrypanosomal potency while retaining selectivity in vitro. While exhibiting submicromolar potency (IC_50_ < 1 μM) on the *T. brucei rhodesiense* bloodstream trypomastigotes, the two diastereomers, **2** and **3**, were also found to be respectively 43- and 80-fold less toxic on L6 mammalian cells. However, on the same species of trypanosomes, the hydroxy-containing analogue, **1**, was only weakly active, highlighting how critical to potency the 2-*E*-octenoic acid ester functionality is in these decahydroquinoline alkaloids [59]. Also possessing submicromolar potency (IC_50_ = 0.6 μM) against the mammalian stage of *T. brucei rhodesiense* is fascaplysin **4** (Figure 2), a quaternary indole alkaloid isolated from the marine sponge *Hyrtios erecta*. Regrettably, this indole alkaloid was characterized by significant cytotoxicity on L6 cells [60]. Also from a marine perspective, Copp and co-workers [61] have reported the isolation of two pyridoacridone alkaloids, ascididemin (**5**), and 2-bromoascididemin (**6**) (Figure 2). Being a pentacyclic DNA intercalating agent, compound **5** was associated with substantial cytotoxic activities. Although the two compounds were, generally, more cytotoxic to macrophages than to *T. brucei rhodesiense*, medicinal chemistry derivatization approaches identified very potent analogues with improved safety profiles, which are discussed in later sections of this review.

##### Peroxides and Saponins

Other marine organisms have furnished highly trypanocidal peroxides. Phytochemical investigation of the marine sponge *Plakortis cfr. Lita* has unraveled an ultrapotent peroxide derivative, manadoperoxide B (**7**, Figure 2) [62]. Evaluation against *T. brucei rhodesiense* revealed that **7** was highly trypanocidal (IC_50_ = 0.009 μM) and was equipotent to the reference drug melarsoprol (IC_50_ = 0.008 μM). Although less potent than manadoperoxide B, other manadoperoxides still retained marked submicromolar potency. These include manadoperoxide I (**8**, IC_50_ = 0.173 μM), and manadoperoxide K (**9**, IC_50_ = 0.198 μΜ). Excitingly, the metabolites were found to be non-toxic on the human cell line HMEC-1. In the same study, Chianese and colleagues were able to draw some structure-activity relationship (SAR) profile around the peroxide **7** by comparing the obtained antitrypanosomal data with that reported in literature for related dioxane derivatives. Close examination of the data revealed that subtle modifications such as positional change of the methyl group on the dioxane ring can drastically influence antitrypanosomal potency [62].

Steroidal saponins from marine sources have also been found to strongly inhibit *Trypanosoma* parasites. A noteworthy example is pandaroside G methyl ester (**10**, Figure 2) isolated from a Caribbean sponge *Pandaros acanthifolium*. Although this metabolite lacks selectivity against mammalian L6 cells (IC_50_ = 0.22 μM), it was found to be highly trypanocidal with submicromolar potency against *T. brucei rhodesiense* (IC_50_ = 0.038 μM) [63]. On the other hand, the oroidin dimer dibromopalau’amine (**11**, Figure 2) isolated from *Axinella verrucosa* has demonstrated submicromolar activity (IC_50_ = 0.8 μΜ, *T. brucei rhodesiense*) with favourable selectivity, selectivity index of 10, against mammalian L6 cells [64,65].

#### 2.3.2. Antitrypanosomal Natural Products from Plant and Microbial Sources

##### Phenolic Natural Products

**Figure 3:**
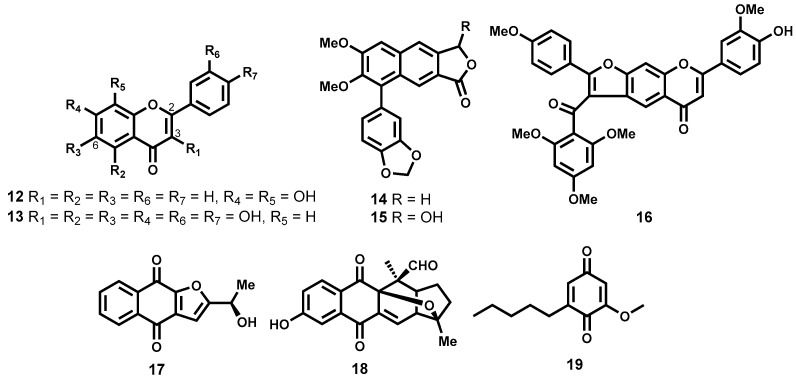
Trypanocidal phenolic and quinone derivatives.

In another study that evaluated 132 flavonoids from different subclasses (flavone, flavonol, flavanone, isoflavone and chalcone subclasses), Räz [66] was able to identify two trypanocidal compounds (Figure 3) which demonstrated submicromolar potency against *T. brucei rhodesiense* while maintaining exceptionally good selectivity: 7,8-dihydroxyflavone (**12**) and quercetagetin (**13**), IC_50_ = 0.16 and 0.8 μM, SI = 1019 and 571 (adenocarcinoma cells (HT-29)), respectively. In addition to the two most potent compounds identified, the minimum structural requirements for potency were also noted. It was generally observed that flavones, a subclass bearing a C2-C3 double bond were more potent than flavanones, another subclass devoid of the double bond at the same position. It was also observed that the flavone derivatives which lack the hydroxyl functionality on C3 were less trypanocidal than hydroxyl-containing flavonols [66].

Another class of phenolic derivatives, the chalcones, have been explored as trypanocidal agents. In one study, β-oxygenated chalcones, Demethylpraecansone B and praecansone B (structures not shown) were isolated from the roots of *Tephrosia aequilata* (Papilionaceae), and were found to exhibit modest trypanocidal potency on *T. brucei rhodesiense* [67]. Also noteworthy amongst the trypanocidal phenolic derivatives are two arylnaphthalide lignans, justicidin B (**14**) and its hydroxylated derivative, piscatorin (**15**, Figure 3). When tested on the bloodstream trypomastigotes of *T. brucei rhodesiense*, compound **14** exhibited strong inhibition with submicromolar activity (IC_50_ = 0.55 μM) albeit cytotoxic on different mammalian cell lines. The hydroxylated derivative **15** was on the other hand 11-fold less potent (IC_50_ = 6.1 μM) [68]. Another chalcone-flavone dimer, cissampeloflavone (**16**, Figure 3), isolated from the aerial parts of *Cissampelos pareira* (Menispermaceae) has been shown to exhibit good potency on the *T. brucei rhodesiense* bloodstream stage [69].

##### Quinones

Some compounds from natural sources containing a quinone moiety have been shown to exhibit exceptionally good antitrypanosomal potency. Employing a bioactivity-guided fractionation approach, Moideen and co-workers [70] were able to isolate a highly potent furanonaphthoquinone, **17** along with another notable naphthoquinoid, isopinnatal (**18**, Figure 3) from the stem bark and root bark extracts. The quinone-derivative **17**, was found to exhibit pronounced antitrypanosomal potency in the low IC_50_ double-digit nanomolar range (IC_50_ = 0.045 μM, *T. brucei rhodesiense*), while, albeit less potent than **17**, isopinnatal (**18**) still retained submicromolar activity on *T. brucei rhodesiense* (IC_50_ = 0.73 μM). However, the cytotoxicity profile on KB cell lines for both compounds is relatively high (IC_50_ = 3.9 μM and 14.8 μM for **17** and **18** respectively).

Primin (**19**, Figure 3), another quinone derivative isolated from the leaves of *Miconia lepidota* [71] has been shown to exhibit potent antitrypanocidal activity (IC_50_ = 0.14 μM) on *T. brucei rhodesiense* with moderate cytotoxicity (IC_50_ = 15.4 μM) against mammalian L6 cell lines. When administered intraperitoneally in the *T. brucei brucei* rodent model, primin was ineffective in curing the infection at 20 mg/kg while higher doses proved toxic [72].

##### Terpenes and Other Metabolites

**Figure 4:**
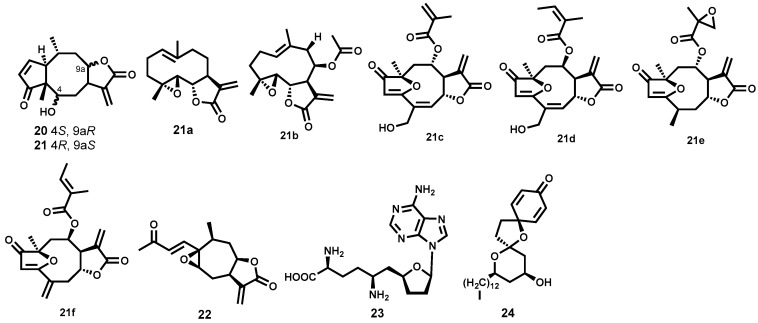
Trypanocidal terpenes and other metabolites.

Two highly trypanocidal sesquiterpene lactones (Figure 4) have been identified among a group of sesquiterpene lactones isolated from *Arnica* and *Inula* species (Asteraceae). Helenalin (**20**) and mexicanin I (**21**) exhibited potent activity against *T. brucei rhodesiense* bloodstream trypomastigotes (IC_50_ = 0.05 and 0.32 μM respectively) while being reasonably devoid of cytotoxicity on mammalian L6 cells (SI = 19.5 and 7.7 for **20** and **21**, respectively). The two diastereomers have a 6-fold difference in potency, a result that shows the importance of stereochemistry for activity in this scenario. The two sesquiterpene lactones have two α,β-unsaturated carbonyl moieties which are potential alkylation sites as Michael acceptors. These sites (cyclopentenone and α-methylene-γ-lactone) can covalently react with various thio groups of various enzymes. The authors observed reduced potency with other sesquiterpene lactones bearing a single alkylation site. It was, therefore, postulated that this class of molecules might be exerting their trypanocidal potency by interfering with trypanothione metabolism, thereby, inducing oxidative stress in the parasite [73]. Another sesquiterpene lactone—parthenolide (**21a**, Figure 4)—with notable trypanocidal potency, has been isolated following a bioassay-guided fractionation of a crude extract from the plant *Saussurea costus* (Asteraceae) [74]. Although **21a** had an unfavorable toxicity profile when evaluated against rat skeletal myoblast L6 cells (IC_50_ = 5.2 μΜ, SI = 6.5), it was found to potently inhibit the bloodstream forms of *T. brucei rhodesiense* (IC_50_ = 0.8 μM). More recently, five highly trypanocidal sesquiterpene lactones isolated from various plant species have been identified [75]. These include **21b**, **21c**, **21d**, **21e**, and **21f** (Figure 4) isolated from *Liriodendron tulipifera* (Magnoliaceae) [76], *Lychnophora diamantinana* [77], *Viguiera robusta* [78], *Eremanthus goyazensis* (Asteraceae) [79] and *Helianthus tuberosus* (Asterceae) [76] respectively. Evaluation against the bloodstream forms of *T. brucei rhodesiense* revealed that **21f** was the most potent (IC_50_ = 0.015 μM) with favorable selectivity (SI = 77). For compounds **21c** and **21d**, it was revealed that the nature of the substituent and stereochemistry at C8 was irrelevant to potency—the two analogues were shown to be equipotent (IC_50_ ~ 0.07 μM). For compounds **21f** and **21e**, however, where the α,β,γ,δ-unsaturated carbonyl system is changed, the impact on potency is quiet significant. In this regard, the loss of one potential reactive site (Michael acceptor) in analogue **21e** (IC_50_ = 0.20 μM) could explain the reduced potency. Compound **21b** also demonstrated a slightly inferior potency (IC_50_ = 0.23 μM) [75]. Although exhibiting inferior potency, it is important to note that many other terpenes and related derivatives isolated from a number of plant species including *Entada abyssinica* (Leguminosae) [80], *Vernonia guineensis* (Asteraceae) [81], *Guarea rhophalocarpa* (Meliaceae) and *Galphimia glauca* (Malphigeaceae) [82,83] have been evaluated against trypanosomes.

Another terpenoid-based metabolite that has shown high in vitro trypanocidal potency is the sesquiterpene lactone, 8-epixanthatin 1β,5β-epoxide (**22**, Figure 4). Evaluation against *T. brucei rhodesiense* revealed strong inhibition of the growth of *Trypanosoma* parasite (IC_50_ = 0.33 μM). It is important to note that the same secondary metabolite has demonstrated notable leishmanicidal potency, which has been discussed in the later section of this review. Moreover, the potent sesquiterpene lactone has been characterized with a relatively favourable toxicity profile on rat myoblast cells [84].

Other classes of natural products derived from microbial sources have shown potential as antitrypanosomal agents. Sinefungin (**23**, Figure 4) produced by *Streptomyces grizeolus* and *S. incarnates* is a natural nucleoside that has demonstrated potent in vitro activity against *T. brucei rhodesiense* bloodstream trypomastigotes in the subnanomolar range (IC_50_ = 0.0004 μM) with a very high selectivity index (SI > 10^6^) [85]. When progressed further to in vivo studies, **23** was found to cure mice infected with *T. brucei brucei*, *T. congolense* and *T. vivax* when administered intraperitoneally [86,87]. However, the compound could not be developed further due to unexpected severe nephrotoxicity in goats at subcurative doses [88]. Also notable are aculeatins, another class of compounds isolated from the rhizome of *Amomum aculeatum* (Zingiberaceae). These are exemplified by aculeatin D (**24**, Figure 4) which exhibited strong albeit nonselective potency against bloodstream trypomastigotes of *T. brucei rhodesiense* (IC_50_ = 0.5 μM and 0.9 μM on *T. brucei rhodesiense* and KB cells respectively) [89,90].

#### 2.3.3. Semisynthetic Antitrypanosomal Natural Products

**Figure 5:**
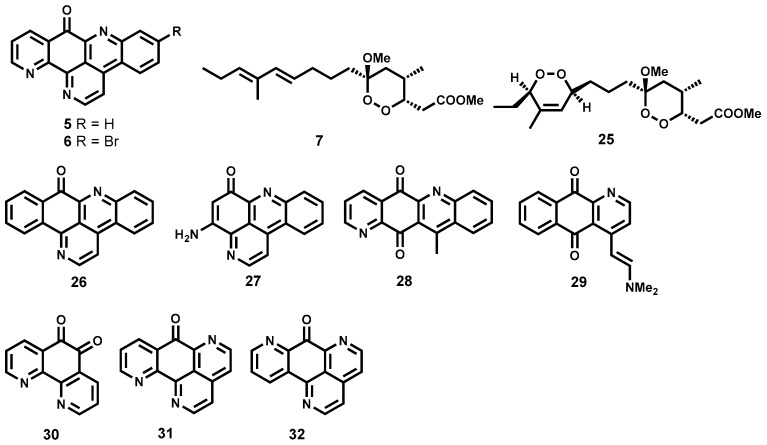
Trypanocidal semisynthetic compounds based on natural product scaffolds.

A number of semisynthetic compounds based on natural product scaffolds have been reported to possess noteworthy trypanocidal potency. A recent example is the semisynthetic analogue **25** (Figure 5), derived from a very potent peroxide manadoperoxide B (**7**, Figure 5, see also Figure 2) originally isolated from a marine sponge *Plakortis cfr. Lita* [62,91]. The semisynthetic analogue demonstrated marked trypanocidal activity against the blood stream forms of *T. brucei rhodesiense* in the submicromolar range (IC_50_ = 0.43 μM) while displaying appreciable selectivity against the L6 cells derived from rat skeletal myoblasts (IC_50_ = 33.82 μM) [91].

Based on two naturally isolated pyridoacridone alkaloids, ascididemin (**5**), and 2-bromo-ascididemin (**6**, Figure 5, see also Figure 2), Copp and co-workers have synthetically generated derivatives that have demonstrated pronounced trypanocidal activity against *T. brucei rhodesiense* [61]. The pyridoacridone analogue **26** (Figure 5), was found to be the most potent (IC_50_ = 0.007 μM) of all the analogues synthesized. Despite exhibiting slightly inferior potency, other derivatives still retained submicromolar potency: **27** (IC_50_ = 0.283 μM), **28** (IC_50_ = 0.018 μM), **29** (IC_50_ = 0.075 μM), **30** (IC_50_ = 0.109 μM), **31** (IC_50_ = 0.064 μM) and **32** (IC_50_ = 0.064 μM). Other derivatives reported in this study were also associated with pronounced potency [61]. Generally, the synthesized derivatives were less cytotoxic on the L6 mammalian cell line compared to the naturally isolated metabolites **5** and **6**. It has been speculated that the mode of action for this class of compounds may involve DNA intercalation along with DNA oxidative damage [61].

## 3. Leishmaniasis

### 3.1. Background of the Disease

Leishmaniasis is a parasitic disease caused by protozoan *Leishmania* parasites and transmission to humans is facilitated by the bite of infected female phlebotomine sandflies. The disease pathology takes three main forms: visceral leishmaniasis (VL, also known as kala-azar), cutaneous leishmaniasis (CL), and mucocutaneous leishmaniasis (MCL) [92]. *Leishmania* parasites are known to be transmitted by over 90 sandfly species. During a blood meal on infected humans or other terrestrial animals, the parasite enters the sandfly where it exists as an extracellular flagellated promastigote in the midgut of the vector. The parasite later develops into an infectious metacyclic promastigote, which is later inoculated into the skin of the host in the next blood meal. The parasites, then, lose their flagella as they transform into intracellular amastigotes upon internalization into macrophages, dendritic cells or neutrophils. Once in the phagolysosomes, the amastigotes can persist and reproduce via binary fusion [93]. The amastigotes released from the lysis of infected phagocytotic cells then invade either additional tissue macrophages or extensively infect the reticulo-endothelial system depending upon the species of the *Leishmania* parasite. The *Leishmania* species, *L. major* and *L. tropica* are the primary causes of CL in the old world while *L. braziliensis* and *L. mexicana* are responsible for CL infections in the new world [94]. MCL is principally caused by *L. braziliensis* although additional species (*L. amazonensis*, *L. panamensis* and *L. guyanensis*) have also been described [95]. In the Indian subcontinent, Asia and East Africa, VL is caused by *L. donovani* while *L. infantum* is responsible for infections in Europe, North Africa and Latin America. The clinical outcome of the disease is influenced by the pathogenic species, as well as the state of host immunity. If the immune response has the capacity to fight the infection, the skin lesions associated with CL are often self-healing with concomitant development of life-long resistance to reinfection [96]. In the case of immunity failure, the disease becomes chronic, with infection progressing to the eticulo-endothelial system resulting in VL which is systemic and non-healing [97]. It is important to note that other parasite species that are responsible for self-healing lesions of the disease can also cause visceralizing infections.

If left untreated, VL can lead to death in over 95% of cases. Common symptoms for VL include irregular bouts of fever, weight loss, enlargement of the spleen and liver as well as anaemia [92]. In the Indian sub-continent as well as East Africa, the disease is highly endemic. On a global scale, the WHO estimates that 200,000 to 400,000 new cases of VL occur each year. Of the new cases reported in 2014, 90% of the cases occurred in 6 countries: Brazil, Ethiopia, India, Somalia, South Sudan and Sudan [92].

CL is characterized by skin lesions, which are largely ulcers on exposed parts of the body, resulting in life-long scars and severe disability. It is the most common version of leishmaniasis with approximately 95% of the cases occurring in the Americas, the Mediterranean basin, the Middle East and Central Asia. Six countries (Afghanistan, Algeria, Brazil, Colombia, Iran, and the Syrian Arab Republic) account for over two thirds of new CL cases [92]. Between 0.7 million and 1.3 million new cases are reported globally every year [92]. A partial or total destruction of nose, mouth and throat mucous membranes can result from infection with MCL. Bolivia, Brazil and Peru account for nearly 90% of the cases [92].

### 3.2. Current Chemotherapy and Associated Challenges

**Figure 6:**
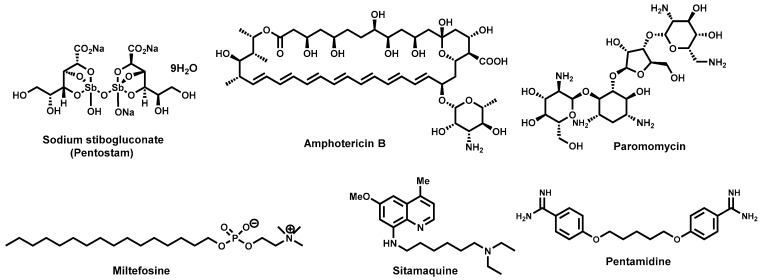
Currently used antileishmanial drugs.

The antimony-containing drugs, also known as the pentavalent antimonials, are the drugs of choice for first line treatment of Leishmaniasis where resistance has not been reported [98]. These include the generic sodium stibogluconate (pentostam, Figure 6), the branded meglumine antimoniate (structure remains a subject of debate) which have been in use for over five decades. Regrettably, the *Leishmania* parasites have become increasingly resistant to the pentavalent antimonial drugs and this brought into question their use in disease-endemic areas [99]. Because antimonials are administered intravenously or intramuscularly, they are not convenient for patients. They are also associated with side effects, which include chemical pancreatitis, elevations in serum aminotransferases and electro-cardiographic abnormalities [40].

Second-line drugs include amphotericin B (Figure 6), which is used in areas where antimonial resistance is common [100]. It exhibits strong binding to ergosterol, the main sterol of fungal and leishmanial cell membranes. Regrettably, amphotericin B is toxic [101,102] despite its high efficiency. Other formulations of amphotericin B have largely circumvented the adverse effects although some are quiet expensive [103]. Other second-line antileishmanial chemotherapeutics include miltefosine (Figure 6), an alkylphosphocholine, originally developed as an anticancer agent. It is the first orally administered drug for the treatment of VL. Having proved its remarkable efficacy in clinical trials [104,105], miltefosine was considered a major breakthrough in antileishmanial chemotherapy [106,107]. However, serious concerns of its teratogenicity may limit its use, it has also been thought that its long half-life (152 h) could encourage the emergence of drug resistance [108]. Paromomycin (Figure 6), another second line anti-leishmanial drug, is chemically an aminogycosidic compound which cures both VL and CL albeit its scarcity has hampered its use in endemic regions [109,110]. Sitamaquine (Figure 6), the only drug originally developed to treat VL, is an 8-aminoquinoline, which also offers an advantage of oral administration. Its efficacy and tolerability was demonstrated in a phase II clinical trial in India [111]. Its efficacy has also been reported in a Kenyan clinical trial [112]. However, side effects including vomiting, dyspepsia, cyanosis, nephritic syndrome, glomerulonephritis, abdominal pain, headache and kidney dysfunctioning were observed in both clinical trials. Pentamidine (Figure 6, also used to treat trypanosomiasis), an aromatic diamine has also been used to treat antimonial-refractory VL patients although its declining efficacy has led to its withdrawal from the market [113].

### 3.3. Antileishmanial Natural Products

The current challenges associated with current chemotherapeutic interventions for Leishmaniasis warrant intensive research efforts into novel antileishmanial therapies. In this section, we review natural products that have demonstrated pronounced antileishmanial potency. Once again, we focus our efforts only on those isolated and characterised natural products that display submicromolar (IC_50_ < 1 μM) potency and those that have shown promising in vivo efficacy.

#### 3.3.1. Antileishmanial Natural Products from Plant Sources

##### Flavonoids, Sterols, Chalcones, Coumarins, Tannins and Aurones

**Figure 7:**
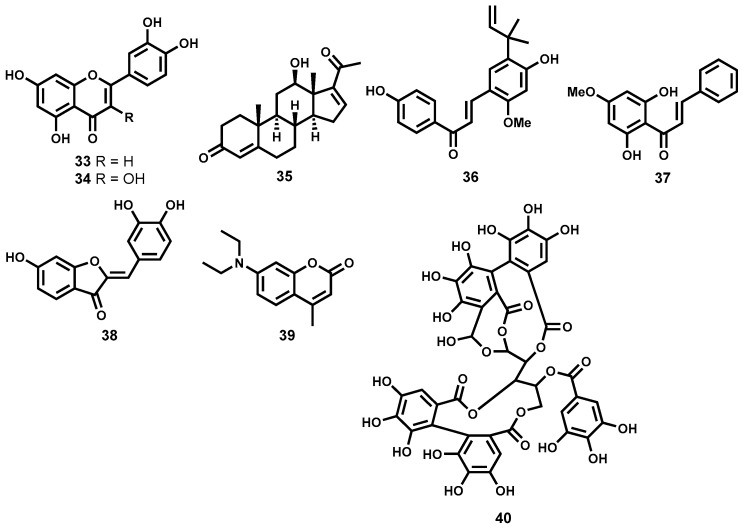
Antileishmanial flavonoids, sterols, chalcones, coumarins, tannins and aurones.

Despite showing a somewhat modest in vitro activity, the flavonoids, luteolin (**33**) and quercetin (**34**, Figure 7) displayed impressive in vivo efficacy. Luteolin showed up to 80% splenic parasite load reduction in infected rodents at a dose of 3.5 mg/kg body weight while quercetin showed a 90% parasite reduction (dose: 14 mg/kg) in the same rodent model. Although quercetin appeared to induce cell cycle arrest in normal human T-cells, such toxicity was not observed for luteolin [114].

In another study, phytochemical investigation of a Mexican plant, *Pentalinon andrieuxii*, led to the isolation of six sterols with remarkable leishmanicidal potency. Of the six isolated sterol derivatives, 6,7-dihydroneridienone (**35**, Figure 7) displayed a combination of high leishmanicidal potency (IC_50_ = 0.03 μM, *L. mexicana*) and negligible cytotoxicity on health bone marrow macrophages from C57BL/6 mice [115].

An oxygenated chalcone, licochalcone A (**36**, Figure 7), isolated from the Chinese liquorice *Glycyrrhiza* spp. (Fabaceae) also exhibited moderate in vitro potency against *L. major* and *L. donovani* promastigotes and amastigotes [116,117] while in vivo efficacy in hamsters showed splenic and liver parasite load reduction of up to 96% (20 mg/kg body weight × 6 days) [118]. When evaluated for cytotoxicity against human leucocytes, lymphocytes and monocytes, no cytotoxic effects were observed. Licochalcone A and related chalcones are known to exert their anti-leishmanial effect by destroying the mitochondrial ultrastructure and are capable of strongly inhibiting the enzyme fumarate reductase in *L. major* [119]. Other chalcone derivatives have been found to demonstrate antileishmanial efficacy comparable to some first-line antileishmanial drugs. For instance, when formulated with polylactide nanoparticles (PLA), the 2′,6′-dihydroxy-4′-methoxy-chalcone (DMC, **37**, Figure 7), isolated from *Piper aduncum* (Piperaceae), reduced cutaneous ulcers by 60% in infected BALB/c mice (440 μg of DMC-PLA × 42 days), an effect that is comparable to that of the first-line drug, glucantime^®^ (meglumine antimoniate) at equivalent doses [120]. The nitrosylated version of DMC also demonstrated efficacy comparable to the first-line drug pentostam^®^ when administered intralesionally in mice [121]. Another interesting group of metabolites that are structurally isomeric to flavones are the aurones. Aurones have demonstrated antileishmanial potency against a wide spectrum of *Leishmania* species albeit also toxic on bone marrow-derived macrophages. Interestingly, an aurone (**38**, Figure 7) devoid of toxicity while exhibiting antileishmanial potency (EC_50_ = 0.33–0.40 μM) across different promastigote *Leishmania* species has been identified [122].

Impressive leishmanicidal potency has also been observed for coumarins, another class of polyphenolic compounds, isolated from various plant species [123,124,125,126]. A typical example of such coumarins, compound **39** (Figure 7) isolated from *C. brasiliense*, has proved highly efficacious when administered intramuscularly at a dose of 18 mg/kg/day or 0.2% topically in *L. amazonensis* infected mice. Interestingly, when administered intramuscularly, its efficacy was shown to be comparable to the standard drug, glucantime^®^ (meglumine antimoniate) [123].

Tannins represent another group of polyphenolic secondary metabolites that exhibit a broad spectrum of biochemical and pharmacological properties [127,128]. Kolodziej et al., have identified a potent leishmanicidal tannin, casuarinin (**40**, Figure 7) among 27 different tannins isolated from *Punica granatum*, *Casuarina*, and *Stachyurus* species. Casuarinin was found to be highly leishmanicidal on *L. donovani* parasites with EC_50_ of 0.52 μM with minimal toxicity on murine host cells (EC_50_ > 26.74 μM) [129,130].

##### Iridoids, Quinones, and Quinoline Alkaloids

**Figure 8:**
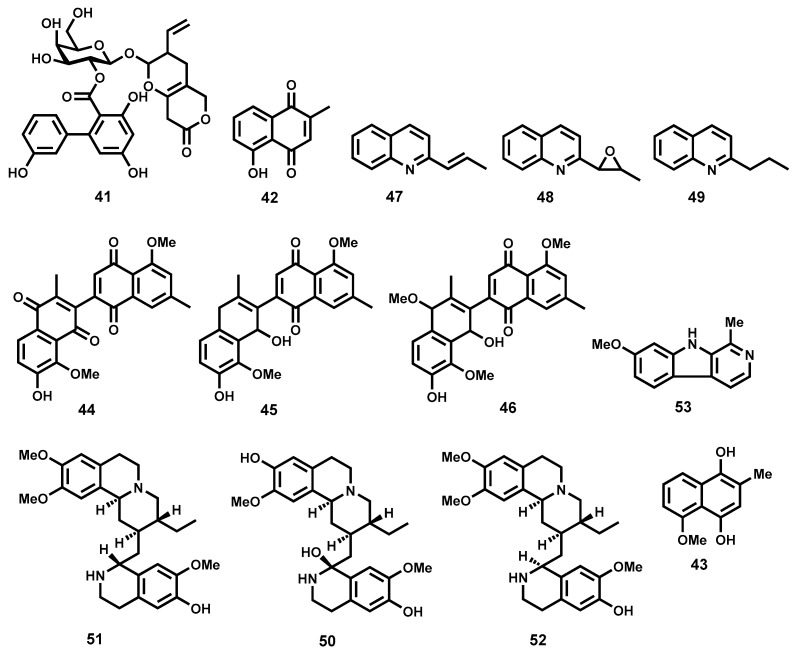
Antileishmanial iridoid, naphtoquinone, quinolines, and alkaloids.

Isolated from *Swertia chirata* (Gentianaceae), amarogentin (**41**, Figure 8), is a secoiridoid glycoside which is a potent topoisomerase I inhibitor and is known to exert its antileishmanial activity by interfering with the formation of the binary complex between DNA and the enzyme [131]. Medda and co-workers [132] were further able to demonstrate the impressive in vivo efficacy of free amarogentin, liposomal as well as niosomal formulations. When evaluated in the hamster model infected with *L. donovani*, the niosomal formulation reduced the splenic parasite load by 90% at a dose 2.5 mg/kg × 6 days. Toxicity, as assessed by blood pathology, histological tissue staining, and levels of certain liver enzymes, has not been observed [132].

Noteworthy antileishmanial properties have also been observed with naphthoquinones, a special class of quinones possessing a naphthyl nucleus. A case in point is plumbagin (**42**, Figure 8), a naphthoquinone isolated from a Bolivian plant *Pera benensis* (Euphorbiaceae). Metabolite **42** displayed remarkable inhibition of *L. donovani* promastigotes (IC_50_ = 0.21 μM) [133,134]. Plumbagin has also been shown to interfere with development of *L. amazonensis* and *L. venezuelensis* in vivo. Additionally, at a much lower dose than that of the first-line drug, glucantime^®^ the plumbagin dimer, 8,8′-biplumbagin has been shown to locally treat localized single lesions [135]. Plumbagin has been shown to promote DNA cleavage mediated by topoisomerase II [136]. A synthetic derivative of plumbagin, the hydroquinoid **43** (Figure 8) has also displayed submicromolar antileishmanial inhibitory activity (IC_50_ = 0.33 μΜ, promastigote forms of *L. donovani*) [134]. Other naphthoquinones with noteworthy leishmanicidal potency include burmanin A (**44**), B (**45**), and C (**46**) (Figure 8), all isolated from *Diospyros burmanica*. Burmanin A (**44**), was found to display the most potent inhibition of *L. major* in the low double digit nanomolar range (IC_50_ = 0.053). Burmanin B (**45**) and C (**46**), exhibited slightly inferior potency to burmanin A. However, their potencies were still in the submicromolar range, IC_50_ = 0.18 μΜ, and 0.15 μΜ respectively [137]. Primin (**19**, Figure 3), a quinone isolated from the leaves of *Miconia lepidota* and known to strongly inhibit trypanosomes (see Section 2.3.2), has also been associated with strong antileishmanial potency against *L. donovani* (IC_50_ = 0.71 μM) while possessing only moderate cytotoxicity (IC_50_ = 15.4 μΜ) on mammalian cells (L6 cell line) [71,72]. Other natural product-based quinones, including pendulone, possessing impressive antileishmanial potencies, have been reported [138].

Quinoline alkaloids have also shown promise as antileishmanial agents exhibiting notable in vivo efficacy in different *Leishmania* disease models. Chimanine B (**47**, Figure 8) and chimanine D (**48**, Figure 8) which are 2-substituted quinolines isolated from *Galipea longiflora* (Rutaceae) have demonstrated strong therapeutic efficacy against experimental CL and VL. When administered to *L. amazonensis* infected BALB/c mice (50 mg/kg body weight × 5 injections at intervals of 4 days), chimanine B reduced the parasite load by 90% while the lesion weight was reduced by 74% [139]. Chimanine D also exhibited substantial parasite reduction (87%) when administered at a dose 100 mg/kg body weight for 5 days. Furthermore, a dihydro version of chimanine B, 2-*n*-propylquinoline (**49**, Figure 8) almost eliminated the liver parasite load (99.9% reduction in an experimental VL model; 94 mg/kg body weight × 10 days; oral administration) [140]. Phytochemical exploration of the tropical plant *Psychotria klugii* by Muhammad and colleagues [141] has revealed highly potent antileishmanial alkaloids. In this study, klugine (**50**), cephaeline (**51**), and isocephaeline (**52**) (Figure 8) were isolated and profiled for leishmanicidal potency. When evaluated against *L. donovani* parasites, cephaeline (**51**) was found to be >20- and >5-fold more potent (IC_50_ = 0.06 μM) than the standard drugs pentamidine and amphotericin B, respectively. On the other hand, klugine (**50**), demonstrated slightly inferior potency (IC_50_ = 0.85 μΜ) to cephaeline. Submicromolar potency (IC_50_ = 0.96 μM) was also observed for isocephaeline (**52**). Compound **51** also proved to be cytotoxic against SK-MEL, KB, BT-549, and SK-OV-3 human cancer cells while **50** was devoid of such cytotoxicity [141].

Alkaloids of the β-carboline amine class have also been shown to potently inhibit the growth of leishmania parasites. These are exemplified by harmine (**53**, Figure 8), an alkaloid isolated from *Peganum harmala* [142]. Harmine’s impressive in vitro antileishmanial potency prompted in vivo evaluation in hamster models. In this study [142], Lala and co-workers subcutaneously administered harmine (**53**) in free form as well as three vesicular forms—liposomes, niosomes and nanoparticles. At a dose of 1.5 mg/kg body weight administered 6 times for 15 days, the free, liposomal, niosomal, and nanoparticular forms of harmine reduced splenic parasitemia by 40%, 60%, 70% and 80% respectively. Excitingly, only marginal nephrotoxicity and hepatotoxicity were observed for vesicular forms of harmine. Due to substantial efficacy against intracellular parasites coupled with a clean hepatotoxicity and nephrotoxicity profile, the vesicular forms of this alkaloid may be considered for clinical application in humans [142]. Regrettably, DNA-binding-related toxicity is well known for harmine and its analogues [143].

##### Saponins

**Figure 9:**
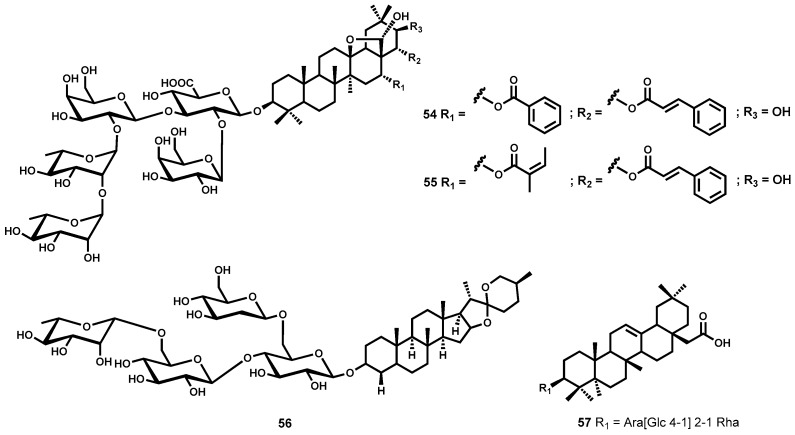
Anti-leishmanial saponins.

Among this class of natural products, some noteworthy highly leishmanicidal metabolites are the two saponins, maesabalides III (**54**) and IV (**55**) (Figure 9) which were isolated from the Vietnamese medicinal plant *Maesa balansae* (Myrsinaceae). Both secondary metabolites exhibited single digit nanomolar range IC_50_ values, 0.005 and 0.009 μM, respectively against *L. infantum* intracellular amastigotes [144]. These two compounds were, interestingly, found to be more potent than the clinically used antileishmanial drug, sodium stibogluconate. When evaluated against human fibroblast (MRC-5) cell line, cytotoxicity was not observed. In vivo investigation showed that a single subcutaneous dose of 0.2 and 0.4 mg/kg for **54** and **55** respectively reduced liver amastigote parasitemia by 90% one week post dosing in the BALB/c mouse model. Another saponin of the steroidal class racemoside A (**56**, Figure 9), isolated from *Asparagus racemosus* (Liliaceae), has shown remarkable potency against *L. donovani* amastigotes with submicromolar activity (IC_50_ = 0.15 μM). Even up to a concentration of 10 μg/mL in murine macrophages, cytotoxicity was not observed [145]. Delmas and colleagues [146] also reported isolation of three saponins from ivy *Hedera helix* (Araliaceae) with notable antileishmanial activity. Hederacholchiside A_1_, **57** (Figure 9) was the most potent exhibiting strong double digit nanomolar range activity (IC_50_ = 0.053 μΜ), against *L. infantum* intracellular amastigotes albeit moderate toxicity on human monocytes was also observed [146].

##### Lignans, Taxoids, Terpenes, Anthranoids and Miscellaneous Antileishmanial Natural Products

**Figure 10:**
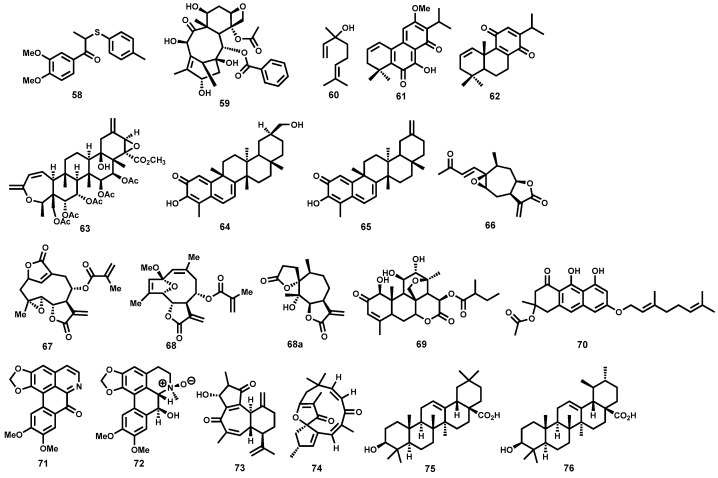
Lignans, taxoids, anthranoids, terpenes and other leishmanicidal metabolites.

Isolated from *Haplophyllum bucharicum* (Rutaceae), the lignan, diphyllin (**58**, Figure 10) has been shown to strongly inhibit (IC_50_ = 0.2 μΜ) the growth of *L. infantum* intracellular amastigotes while only moderately inhibiting promastigotes of the same parasite species. Diphyllin has, however, been shown to be antiproliferative on human monocytes [147]. Taxoid compounds like paclitaxel, isolated from *Taxus baccata* (Taxaceae) are well known in anticancer therapy. From an antileishmanial perspective, another taxoid compound, 10-Deacetylbaccatin III, **59** (Figure 10), isolated from the same plant, has demonstrated strong inhibition of the growth of intracellular *L. donovani* amastigotes with an IC_50_ value of 0.07 μM. Cytotoxic evaluation showed that the compound was well tolerated in macrophages up to a concentration of 5 μM. The mode of action for this taxoid compound has been thought to involve stimulation of nitric oxide production in macrophages and not inhibition of microtubule depolymerisation, which is the mechanism of action of paclitaxel in cancer cells [148]. Impressive leishmanicidal activity has been observed for the monoterpene linalool (**60**, Figure 10) isolated from a plant *Croton cajucara* (Euphorbiaceae). It has shown strong antileishmanial activity against *L. amazonensis* promastigotes and intracellular amastigotes—LD_50_ = 0.028 and 0.14 μM respectively. Exposure of preinfected macrophages to 15 ng/mL of linalool-rich essential oil was found to discourage macrophage-parasite interaction while nitric oxide production was stimulated. Both promastigotes and intracellular amastigotes were completely destroyed upon exposure for 1 h while no cytotoxicity to murine macrophages was observed [149].

Other notable terpene metabolites that have demonstrated strong leishmanicidal potency include two diterpenes: 7-hydroxy-12-methoxy-20-nor-abieta-1,5(10),7,9,12-pentaen-6,14-dione (**61**) and abieta-8,12-dien-11,14-dione (**62**) (Figure 10), both isolated from the roots of *Salvia cilicica* (Lamiaceae). Both diterpene derivatives, **61** and **62**, exhibited strong leishmanicidal potency against intracellular amastigote forms of both *L. donovani* (IC_50_ = 0.17 and 0.12 μM, respectively) and *L. major* (IC_50_ = 0.29 and 0.18 μM, respectively) [150]. In another study, Danelli and co-workers [151] have reported a strongly leishmanicidal nor-triterpene, **63** (Figure 10), isolated from *Lophanthera lactescens*. The metabolite, **63**, strongly inhibits the amastigote forms of *L. amazonensis* with an IC_50_ value of 0.5 μΜ. Two other terpenoid metabolites, isoiguesterin (**64**) and its analogue, 20-epi-isoiguesterinol (**65**) (Figure 10), both isolated from *Salacia madagascariensis* (Celastraceae), have strong submicromolar leishmanicidal potency. Isoiguesterin (**64**) was highly leishmanicidal against both *L. donovani* and *L. mexicana*—IC_50_ = 0.20 and 0.082 μM, respectively. Evaluation of analogue **65** against *L. donovani* revealed that it was almost three-fold more potent (IC_50_ = 0.079 μM) than metabolite **64** [152].

A sesquiterpene lactone, 8-epixanthatin 1β,5β-epoxide (**66**, Figure 10) isolated from the Sudanese plant *Xanthium brasilicum Vell* is another terpenoid metabolite that has displayed noteworthy leishmanicidal in vitro potency against *L. donovani* with a submicromolar IC_50_ value of 0.6 μM. The analogue also exhibited fairly minimal cytotoxicity on rat myoblast cells [84]. Two other sesquiterpene lactones isolated from the Brazilian plant *Elephantopus mollis* have exhibited strong inhibition of *L. major* promastigotes. Elephantopin (**67**) and 2-deethoxy-2β-methoxyphantomolin (**68**) (Figure 10) possessed good potencies (IC_50_ ≤ 0.28 μM) against the extracellular promastigote forms of *L. major* [153]. Furthermore, bioassay-guided fractionation of an organic extract from an Argentine plant, *Ambrosia tenuifolia*, led to isolation of a significantly leishmanicidal sesquiterpene lactone—psilostachyin (**68a**, Figure 10) [154]. When tested against the promastigote forms of *Leishmania* parasites, **68a** displayed submicromolar potency (IC_50_ = 0.43 μM). Excitingly, toxicity evaluation against the T lymphocytic cells showed high selectivity [154].

Simalikalactone D (**69**, Figure 10), also a terpenoid metabolite belonging to the decanortriterpenoid subcategory and isolated from the root bark of *Simaba orinocensis* (Simaroubaceae) has shown impressive leishmanicidal potency. It was found to be highly leishmanicidal on *L. donovani* promastigotes (IC_50_ = 0.07 μΜ). Moreover, under the assay conditions, the decanortriterpenoid metabolite, **69**, was >46- and >31-fold more potent than the two standard antileishmanial drugs, pentamidine and amphotericin B. When further screened for cytotoxicity against Vero cells, **69**, displayed a fairly favourable selectivity (IC_50_ on Vero cells = 4.8 μM). The authors also explored some structure-activity relationships around metabolite **69**. When the two hydroxyl groups on the northern cyclohexyl ring were acetylated, antileishmanial activity was completely eliminated. The Michael acceptor carbonyl group on the western portion of the molecule was also very critical to potency [155].

More recently, Lenta et al., have reported strong antileishmanial potency of an anthranoid, acetylvismione D (**70**) isolated from *Psorospermum glaberrimum* (Clusiaceae), a Cameroonian plant traditionally used for treatment of parasitic diseases. The anthranoid compound exhibited strong in vitro leishmanicidal activity (IC_50_ = 0.09 μM) against *L. donovani*, which was far more superior than the reference drug miltefosine (IC_50_ = 0.46 μM). In vitro cytotoxicity, as tested against mammalian cell lines (L6), was minimal [156]. Dicentrinone (**71**, Figure 10), is one alkaloid among many others successfully isolated from *Duguetia furfuracea* (Annonaceae) which has demonstrated high leishmanicidal potency against promastigote forms of *L. braziliensis* (IC_50_ = 0.01 μM). Promising leishmanicidal potency (IC_50_ = 0.11 μM) against the same *Leishmania* species was also observed for the charged alkaloid, duguetine β-N-oxide (**72**, Figure 10) [157].

Potent antileishmanial terpenoid derivatives have also been isolated from *Jatropha* species [158]. In this regard, phytochemical investigation of *Jatropha grossidentata* led to isolation of a highly potent leishmanicidal diterpene, jatrogrossidione (**73**, Figure 10). In vitro leishmanicidal evaluation, as determined using an IC_100_ value, shows strong potency (IC_100_ = 2.4 μΜ). The Jatropha-derived metabolite was, interestingly, found to be more potent than the standard drugs glucantime and pentamidine against *Leishmania* parasites. When evaluated against the amastigote forms of the *Leishmania* parasites, jatrogrossidione exhibited a submicromolar potency (IC_50_ = 0.8 μM) with toxic concentrations being slightly higher than the IC_50_ value. Jatrophone (**74**, Figure 10), another diterpene isolated from *Jatropha isabellii*, was found to significantly suppress parasitemia in *L. amazonensis* (pH 8) infected BALB/c mice when administered subcutaneously at 25 mg/kg/day for 13 consecutive days. When compared to glucantime administered at a much higher dose (112 mg Sb^V^/kg/day), jatrophone still possessed superior efficacy [158]. Regrettably, such subcutaneous administration of jatrophone proved too toxic, with half of the animals dying during the experiment [158].

In another study and employing a bioguided fractionation approach, Tan and co-workers have reported the isolation and antileishmanial evaluation of two triterpenoic acids [150]. Oleanolic acid (**75**), and ursolic acid (**76**) (Figure 10), both isolated from the roots of *Salvia cilicica* (Lamiaceae), proved highly potent against the amastigote forms of *L. donovani* and *L. major* (IC_50_ = 0.007–0.12 μM). The two metabolites also exhibited marked potency (IC_50_ = 0.051–0.137 μM) against the promastigote forms of *L. donovani* and *L. major*. Cytotoxicity, as measured on non-parasitised macrophage-like RAW 264.7 cells for both **75** and **76** (IC_50_ = 0.13 and 0.016 μM respectively) remains a serious concern.

#### 3.3.2. Antileishmanial Natural Products from Marine Sources

**Figure 11:**
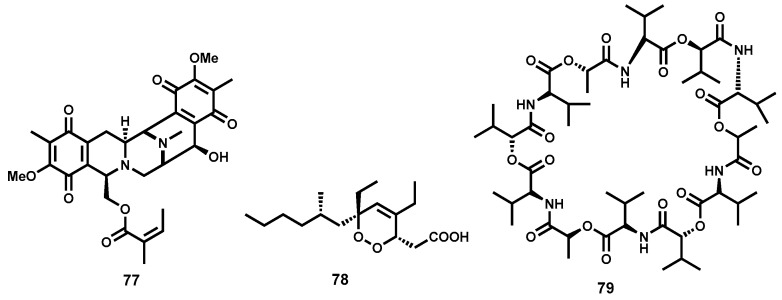
Marine-derived antileishmanial natural products.

Although there seems to be a paucity of information on marine-based isolation of Antileishmanial metabolites, some potent leishmanicidal marine-derived metabolites have been reported. A marine-derived alkaloid, renieramycin A (**77**, Figure 11), isolated from *Neopetrosia* species has been reported to significantly inhibit *L. amazonensis* with an IC_50_ value of 0.35 μM [159]. Furthermore, phytochemical investigation of the Palauan sponge *Plakortis aff angulospiculatus* led to the isolation of a potent cyclic peroxide **78** (Figure 11) with submicromolar activity against *L. mexicana* (LD_50_ = 0.97 μM). At a concentration of 3.4 μΜ, the cyclic peroxide induces cell membrane lysis after 24 h with a drastic decrease in motility after 0.5 h [160]. Marine-based microbes have also inspired natural products that have shown relevance as antileishmanial agents. In this context, and relatively recently, Linington and co-workers have reported the isolation of valinomycin (**79**, Figure 11), from *Streptomyces* strains. Valinomycin was found to be highly potent against *L. major* promastigotes (EC_50_ < 0.11 μM) [161]. Unfortunately, the peptide metabolite exhibited an unfavourable toxicity profile against 293T kidney epithelial cells and J774.1 macrophages [162].

#### 3.3.3. Semi-Synthetic Antileishmanial Natural Products

**Figure 12:**
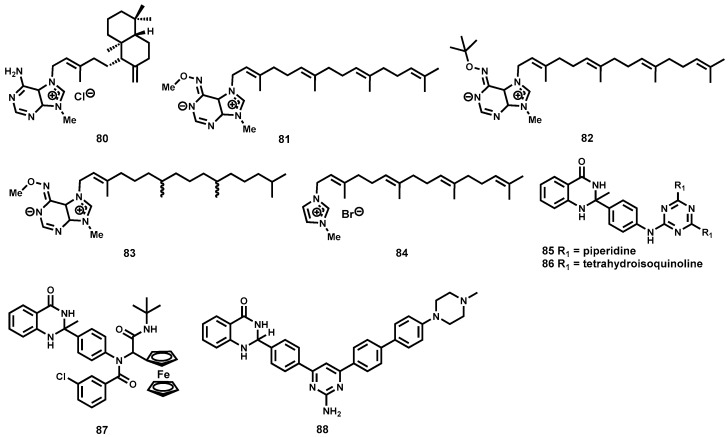
Natural product-based synthetic/semisynthetic antileishmanial compounds.

A limited number of potent antileishmanial semisynthetic compounds inspired by natural product scaffolds have been reported in literature. In a recent study, Vik and co-workers have reported the total synthesis of agelasine D (**80**, Figure 12), a marine natural product originally isolated from the marine sponge *Agelas nakamurai* [163].

Various derivatives of **80** (Figure 12) were also synthesized and evaluated for leishmanicidal activity against amastigote forms of *L. infantum*. Among the most potent derivatives were **81** (IC_50_ = 0.20 μM), **82** (IC_50_ = 0.54 μM), **83** (IC_50_ = 0.50 μM), and **84** (IC_50_ < 0.25 μM). Regrettably, with the exception of analogues **81** and **84**, most evaluated analogues were also highly toxic on MRC-5 fibroblast cells. In another study inspired by quinazolinone-containing natural products with pharmacological relevance, Sharma et al., have synthesised leishmanicidal quinazolinones hybridized with heterocyclic moieties and other substructures [164]. The study identified four significantly potent analogues (**85**, **86**, **87** and **88**, Figure 12) in vitro and in vivo. Although analogues **85** and **86** exhibited moderate potency in vitro, IC_50_ = 3.95 and 4.39 μM, respectively, against intracellular amastigote forms of *L. donovani*, they both possessed pronounced in vivo efficacy (73.2% and 80.9% reduction in parasitemia) in *L. donovani* infected hamster models. The ferrocene-containing analogue **87**, possessed submicromolar potency in vitro (IC_50_ = 0.73 μM) but only moderately efficacious in vivo. Additionally, analogue **88** also demonstrated good potency in vitro (IC_50_ = 0.65 μM) although its unfavourable toxicity profile discouraged further investigation in vivo.

## 4. Schistosomiasis

### 4.1. Background of the Disease

Schistosomiasis, also known as bilharzia, is a parasitic disease caused by flatworms of the genus *Schistosoma*. The public health impact of schistosomiasis is revealed by records showing that the infection is only second to malaria with respect to epidemiology and morbidity among the parasitic diseases [165]. Statistics indicate that close to 260 million people required preventive chemotherapy in 2014, the majority of which are in Africa, Asia and South America [166,167].

Three types of *Schistosoma* species are largely responsible for human schistosomiasis: *Schistosoma mansoni*, *Schistosoma japonicum* and *Schistosoma haematobium* [168]. The human infection occurs when the intermediate fresh water snail releases the larva form known as cercaria that penetrates the skin of humans in contact with the infected water during water-related activities. The larva then transforms and migrates through systemic circulation to lodge in the portal veins where the paired parasites mature and migrate to the mesenteric venules (*S. mansoni* and *S. japonicum*) and urinary bladder plexus (*S. haematobium*) where the female lay eggs. The eggs passed through faeces or in urine, hatch into the ciliated larval form referred to as miracidia in fresh water, infect the appropriate intermediate snail host and transform into the free swimming larva, cercaria, which are then ready to infect the next human, thus completing the cycle [8,169].

Host immune reactions to the schistosome eggs contribute to disease pathology which runs a chronic and debilitating course and is often insidious, with diagnosis only realised after severe organ damage or complications [170]. In the case of intestinal schistosomiasis, gastrointestinal bleeding, obstruction, portal hypertension, hepatomegaly and splenomegaly may arise while with urinary schistosomiasis, haematuria, obstructive uropathy and cancer of the urinary tract are possible outcomes [171]. Moreover, schistosomiasis can worsen prognosis with co-infections and is known to increase HIV/AIDS transmission due to the urogenital pathology by *S. haematobium*, further affirming the public health significance of this parasitic disease [172,173,174].

### 4.2. Intervention Strategies and Challenges

**Figure 13:**
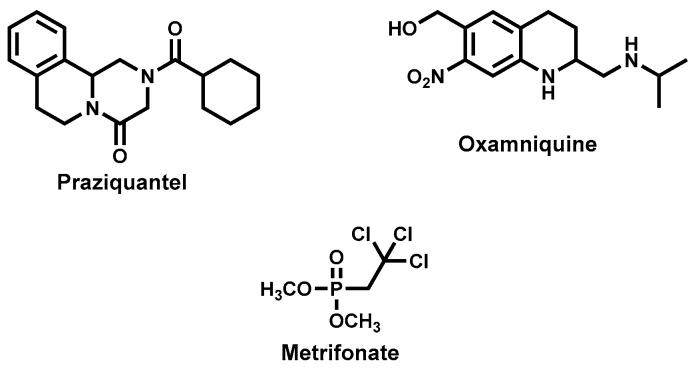
Clinically established antischistosomal drugs.

Approaches to the control of schistosomiasis include snail vector control, preventive chemotherapy and treatment of the disease to control morbidity. For over four decades, praziquantel (Figure 13) has remained the drug of choice for the treatment of all types of human schistosomiasis [175,176]. The other two drugs-oxamniquine and metrifonate (Figure 13) are only singly active against *S. mansoni* and *S. haematobium*, respectively and are no longer preferred for clinical use.

Despite the success and notable advantages with the use of praziquantel that include its low cost and possessing activity against all the three species of schistosomes, there are nonetheless some limitations. The drug is only active against the mature worms and not the newly transformed worms hence cannot prevent disease re-infection and, therefore, repeated drug administration is necessary [177].

Furthermore, commercial praziquantel is a racemate, comprising both the *R* and *S* isomers yet only the (*R*) isomer is responsible for antischistosomal activity. It has a bitter taste and may contribute to drug non-compliance especially in the mass drug administration programs and, importantly, has been reported to have reduced efficacy in some schistosome strains raising worries of development of resistance [178,179,180].

On the basis of the public health impact and the paucity of drug discovery efforts for schistosomiasis, there exists a clear and urgent need for newer drugs as alternatives to praziquantel should the resistance reported in other areas rise or spread to clinically significant levels. In this quest, the antischistosomal effects of natural products have been investigated and several in vitro and in vivo evaluations pursued. Moreover, there are several reports of exploratory clinical trials for schistosomiasis involving drugs of natural product origin.

#### Parameters Assessed

In evaluating the in vitro and in vivo activities of potential antischistosomal agents, important parameters assessed include effects on features that interfere with reproduction ability: worm pairing, worm migration, egg laying, surface changes that impede adherence within the gynaecophoric groove, motility and the ability of the parasites to attach to the host tissues. Among the common morphological features assessed is the tegument, which is vital for the parasite attachment to the host, nutrition and the circumventing of host immune response to the parasite [181,182]. Other observations include comparing pathological changes between controls and treated animals—mainly regarding the liver and spleen- the ability to kill worms, time taken to kill the worms and the reversibility of effects upon withdrawal of the agent under investigation [176].

### 4.3. Examples of Natural Product Leads or Drugs with Antischistosomal Activities

#### 4.3.1. Epiisopilotulorine

**Figure 14:**
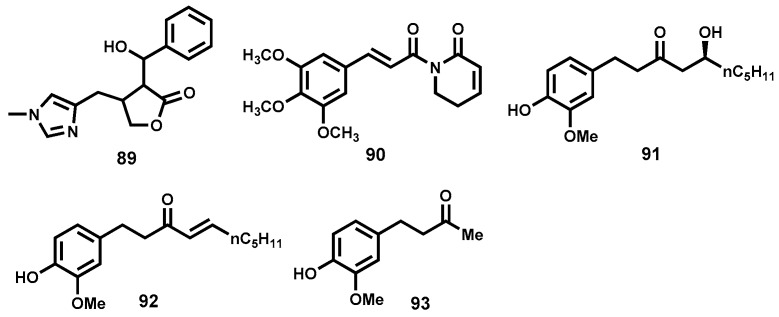
Antischistosomal alkaloids and ginger-derived metabolites.

The imidazole alkaloid from *Pilocarpus microphylus*, epiisopilotulorine (**89**, Figure 14) has been shown to elicit in vitro activity at a concentration of 524 µM. In addition to inhibiting the ability to lay eggs at below lethal dose, the compound had antiinflammatory action, which would be beneficial in resolution of granulomas, a feature of chronic schistosomal infections.

With a safe toxicity profile (530 mg/kg) and accompanying spontaneous resolution of effects; in vivo efficacy studies were pursued at doses of 40, 100 and 300 mg/kg. Epiisopilotulorine displayed inverse dose-response relationship prompting the researchers to propose pharmacokinetics studies of this compound [183].

#### 4.3.2. Piplartine

Piplartine (5,6-dihydro-1-[(2*E*)-1-oxo-3-(3,4,5-trimethoxyphenyl)-2-propenyl]-2(1*H*)-pyridinone **90**, Figure 14), the principal alkaloid from long pepper, *Piper tuberculatum* and other species of the Piperaceae family [184,185], is an amide with an array of activities both against infectious and non-infectious agents, including promising in vitro antischistosomal activity [186,187,188]. At a sublethal concentration of 6.3 µM, egg-laying was inhibited whereas at 15.8 µM, piplartine caused death of worms within a day. In another study [187], piplartine killed 50% of worms after 2 h at a dose of 393.9 µM while it took 24 h at a dose of 12.6 µM to produce the same effect. Praziquantel, used as a positive control in this experiment, produced this effect in 2 h at 10 µM. Other extensive studies reveal the genotoxic potential [189] and low systemic toxicity of piplartine. Further safety profiling carried out on Vero cells and human peripheral blood mononuclear cells [190] failed to show cytotoxicity at the maximum tested concentration at which piplartine is known to be toxic to schistosomes. Mouse pharmacokinetics studies indicate that piplartine has moderate bioavailability and with additional metabolic stability studies, this compound can form a starting point for antischistosomal drug discovery [189,191,192].

#### 4.3.3. Ginger

A rhizome from the Zingiberaceae family, ginger (*Zingiber Officinale*) has numerous culinary and medicinal uses [193,194]. Documented effects, believed to be chiefly owed to the volatile oils Gingerol (**91**), zingerone (**92**) and shogaol (**93**) (Figure 14), range from antiinflammatory, antiarthritic, antidiabetic, hypolipidaemic and hypocholesterolaemic.

In one study, although in vitro activity was demonstrated with the aqueous extract, there was no observed in vivo activity. On the other hand, an organic extract using ethyl acetate dosed at 500 mg/kg resulted in the decrease in worm recovery and egg production [178] and at 200 mg/L killed adult worms [193]. The failure to replicate the observed in vitro effects of the ginger extracts in in vivo studies is not a surprising occurrence in drug discovery and presents an opportunity for medicinal chemistry, formulation or dose optimisation studies in surmounting the possible pharmacokinetics hurdles.

#### 4.3.4. Phytol

**Figure 15:**
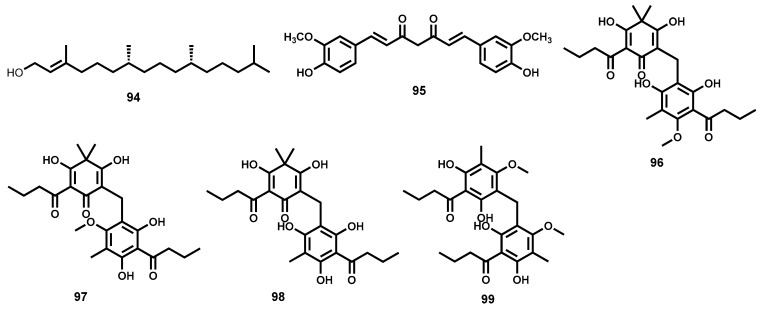
Antischistosomal phloroglucinols, curcumin, and phytol.

The ubiquitous diterpene alcohol 3,7,11,15-tetramethyl-2-hexadecen-1-ol (phytol, **94**, Figure 15), found in chlorophyll, possesses diverse biological and pharmacological activities including immunomodulatory, antiallergic, antinociceptive and antioxidant [42,195,196]. Besides, phytol is frequently used as a food additive, inferring that it is tolerable and hence safe in humans, a pivotal concern in drug discovery programs. In a murine model of schistosomiasis, phytol, at 40 mg/kg dose, killed adult worms while at sublethal doses, it produced changes consistent with antischistosomal activity including: reduced worm motility, decreased egg production and total worm reduction, in a dose dependent manner [195].Since the mechanism of possible toxicity with the use of phytol is predictable [197,198] and that it has a tractable chemical structure, structure-activity and structure-property relationship studies can be readily pursued. Moreover, due to its ubiquitous distribution, costs of production can be projected to be low and this might translate into an affordable drug product, an attractive end point since the disease affects mostly the poor people to whom the cost of medication is out of reach.

#### 4.3.5. Curcumin

A rhizome of the Zingiberaceae family, curcumin (turmeric, **95**, Figure 15), although commonly used as a cooking component, has proven biological activities, including antiprotozoal activity [199,200]. Curcumin has been shown to possess activity against *L. donovani* while Magalhães and colleagues profiled this compound for antischistosomal activity at doses between 5 and 100 µM where a dose-response relationship was obtained [201].

#### 4.3.6. Phloroglucinols

Phloroglucinols from the Dryopteris species elicit antibacterial, antioxidant and antitumour enhancing, vermifuge and antihelminthic activities [202,203]. Principal components associated with the antischistosomal action of phloroglucinols include the structurally related compounds: aspidin (**96**), desaspidin (**97**), flavaspidic acid (**98**) and methylene-bis-aspidinol (**99**) (Figure 15) [202,203,204]. In one in vitro study, whereas aspidin and desaspidin were observed to kill worms at concentrations of between 25 and 100 µM, flavaspidic acid and methylene-bis-aspidinol required higher concentrations of 50–100 µM and 100 µM respectively to elicit the same effect. Interestingly, these compounds appear to induce different morphological effects in the parasite since desaspidin failed to cause decoupling of worms while methylene-bis-aspidinol did not reduce worm viability [205] and may suggest divergent modes of action of these constituent compounds.

#### 4.3.7. Cinchona Alkaloids—Quinine and Derivatives

**Figure 16:**
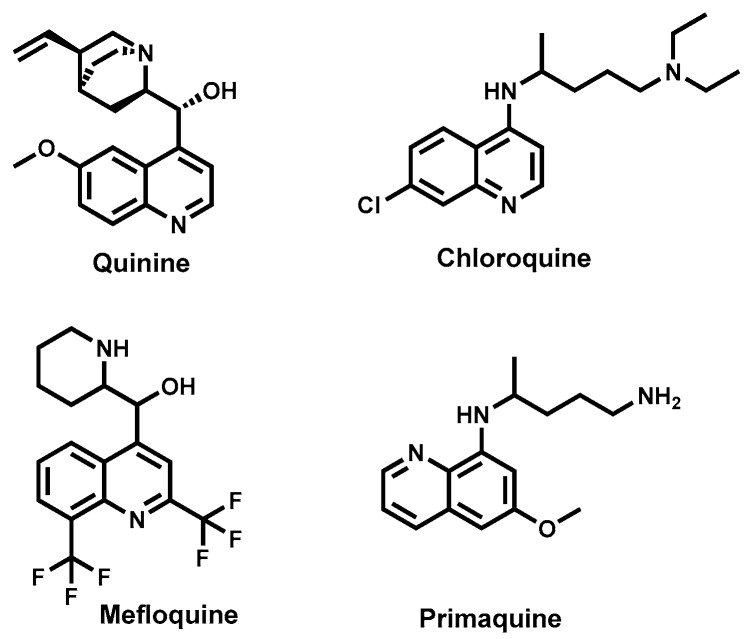
Antischistosomal cinchona alkaloids and derivatives.

The bark of the cinchona tree is a rich source of alkaloids including the antimalarial agent quinine. Chloroquine, a synthetic analogue of quinine was previously successful in the treatment of malaria and its use only decimated with the advent of widespread resistance of plasmodia parasites to its use [206,207]. Together with mefloquine and primaquine, these quinine-based drugs (Figure 16) have been evaluated for their antischistosomal properties, yielding attractive results [208,209,210].

Both in vitro and in vivo studies of mefloquine reveal the ability to reduce the worm burden in infected mice with notable synergy when used together with the artemisinin derivative, artesunate. Mefloquine and primaquine, in a study by Holtfreter and co-workers, showed time- and dose-dependent in vitro antischistosomal activities in the tested concentration range 0.5–2 µg/mL [211]. In an exploratory clinical trial in West Africa, mefloquine produced equal cure rates with artesunate at 21% and 25% respectively and was synergistic with artesunate whereby a three-fold increase in effect was observed (61%) and a 95% reduction in eggs [212]. In this study, the standard treatment for schistosomiasis, praziquantel, proved to be the most effective drug with 88% cure rate. Given the observation that mefloquine is more effective against the immature schistosomulae stage as compared to the adult worms [208,211], combined therapy with praziquantel has been evaluated. As predicted, this combination was synergistic when the two drugs were used in the ratio of their medium effective concentrations [213].

The mechanism of antimalarial action of chloroquine and mefloquine is yet to be fully known. However, it at least consist, in part, of the interference with the haemoglobin degradation pathway where it causes the built up of toxic heme [206,208]. Schistosoma parasite biology contain this pathway also and is a possible drug target in antischistosomal drug discovery [165]. On the other hand, primaquine is known to interfere with mitochondrion function leading to oxidative damage while both primaquine and mefloquine might cause influx of calcium ions into cells leading to spasms [211].

#### 4.3.8. Artemisinins and Derivatives

**Figure 17:**
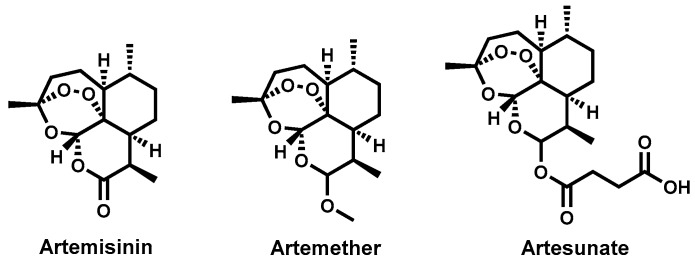
Artemisinin derivatives with antischistosomal potency.

The discovery of the antimalarial properties of artemisinin (Figure 17), a sesquiterpene lactone, from Sweet wormwood, *Artemisia annua* L. (Asteraceae) has revolutionized malaria treatment [214,215,216]. Subsequently, derivatives of artemisinin such as artemether, arteether and artesunate have been developed with improved potency, pharmacokinetics profile and chemical stability and are part of the components of the first line treatment regime in malaria endemic regions [176]. Just as with antimalarial activity, the antischistosomal properties of artemisinins were first reported in China [217]. In that country, artemisinin-derived agents, mainly artemether, were used extensively to control infections in the regions where *S. japonicum* infections were endemic [167]. Studies have followed in other geographical regions and including tests on other *Schistosoma* species [218].

In a study by Le and co-workers, artemether tested experimentally in dogs or mice decreased worm burden and was effective against the migratory larval forms—schistosomula [217]. Later studies reported that schistosome eggs were unresponsive to artemether treatment [219,220]. Other studies followed confirming antischistosomal properties of other artemisinin derivatives—artesunate [221], arteether [222] and dihydroartemisinin [223]. Artemether produced greater effects against female worms compared to the males and induced hepatic shift in worms from mesenteric venules to the liver [219,220]. Moreover, several studies have confirmed that artemether in vivo is more active against the schistosomulae stage than the adult stage [224,225]; an effect that was not only dose-dependent but also influenced by frequency of administration and the route used whereby the intragrastric route appeared more effective than the intramuscular one [226]. In another study, artemether administered intramuscularly at 50 and 100 mg/kg decreased egg-laying and total worms, particularly females in infections with *S. mansoni*. The liver and spleen of the treated animals were noticed to decrease in weight [167].

Since the artemisinins are already in clinical use, and there is geographical overlap between malaria- and schistosomiasis-endemic regions, exploratory clinical trials have been undertaken to ascertain the clinical efficacy and potential of the artemisinins to prevent transmission and contribute to the treatment of schistosomiasis. In clinical trials carried out in West Africa, artemether reduced the incidence and intensity of infection among the study participants-mostly school-going children [227,228]. Due to the activity of artemether in preventing the maturation of schistosomes, adult worms are prevented from developing and hence egg-laying precluded. This decreases the transmission of the disease as well as pathology due to presence of eggs and the adult worms in the organs. For this reason, artemether has been proposed as a potential chemoprophylactic agent among high risk groups [228,229]. Furthermore, studies comprising combined administration of an artemisinin-derived drugs with praziquantel have been explored [230]. In this approach, synergy is envisaged whereby praziquantel would produce greater activity against the adult stage of the parasites while the artemisinin component would be more active against the schistosomulae. This combined effect should translate into the ability to prevent re-infection; an outcome that is not possible in the present use of praziquantel alone. The benefit of having an antimalarial drug possessing antischistosomal property is that with a single drug administration, two diseases can be targeted [228,231] which is an advantage from an economic point of view as well as compliance to medication, due to the decreased pill burden. However, this benefit has a serious potential downside: the use of an antimalarial drug in schistosomiasis raises concern for the potential selection of resistance by the plasmodia parasites—a dangerous scenario especially in the face of limited range of antimalarial drugs [176,232]. Thus, it has been recommended that artemether might be used safely in areas where schistosomiasis is endemic but where malaria is not, to reduce incidence of infection [176].

Artemisinins are believed to achieve their toxicity to plasmodia parasites, as well as to schistosomes, due to the labile endoperoxide group which cleaves to generate reactive oxygen species that interact with biomolecules within the parasite leading to parasite death [233,234]. Medicinal chemistry optimisation of related chemical classes that incorporate the peroxide moiety such as 1,2,4 trioxanes have been explored and present the artemisinins as viable templates for lead development [235].

#### 4.3.9. Allicin

**Figure 18:**
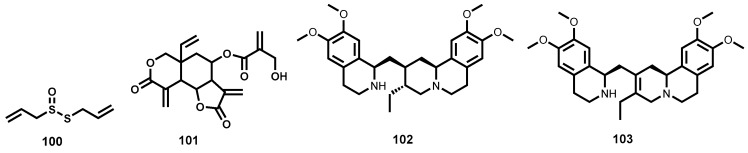
Structures of allicin, vernodalin, emetine and dehydroemetine.

Garlic (*Allium sativum*; Liliaceae) possesses numerous anti-infective properties, among them, antiparasitic [236]. Allicin (**100**, Figure 18), a simple molecule comprising of an unsaturated aliphatic system is identified as the major component and is responsible for the reported antischistosomal activity of garlic. Lima et al., 2011 reported on the effect of incremental doses of allicin (up to 20 mg/mL) on the tegument of adult worms. This effect was dose-dependent and, higher than therapeutic concentrations were required to produce detrimental effects [237]. In earlier findings, Riad and colleagues reported on the curative parasitological effects, against *S. mansoni*, of garlic extract at 50 mg/kg. Interestingly, the observed antischistosomal effects were reversed at a higher dose of 100 mg/kg [238,239].

#### 4.3.10. *Vernonia amygdalina*

The tropical plant *Vernonia amygdalina* (Asteraceae) is a rich source of sesquiterpene lactones and steroidal glucosides with their corresponding aglycones [240], which have been investigated for antischistosomal activity. In the study led by Jisaka, despite failing to produce significant effects at 2 ppm—the concentration at which praziquantel is effective—the sesquiterpene lactones and steroidal glucosides from this plant inflicted changes at 200 ppm with vernodalin (**101**, Figure 18) being most potent, as it produced detrimental effects at 20 ppm [241].

#### 4.3.11. Emetine

Emetine (**102**, Figure 18) is the major alkaloid found in the root of the plant *Cephaelis ipecacuanha* (Rubiciae) with clinical use in amoebiasis and for inducing emesis. Dehydroemetine (**103**, Figure 18) is a closely related analogue resulting from unsaturation of emetine [242,243]. These compounds have been shown to possess antitumour activity and are believed to function via inhibition of protein synthesis [244,245,246]. Evaluation of emetine for antischistosomal activity suggested that double the dose used in amoebic infections would be necessary, an unfavourable profile given the high toxicity of the compound. Dehydroemetine, although possessing a better safety profile than emetine, required prolonged administration—given for 30 days intravenously. These features make the drug expensive for a population that is already impoverished making it less affordable hence not favourable [175,247].

#### 4.3.12. Other Natural Product Leads: Mevinolin (Lovastatin), Plumbagin and Sanguinarine

**Figure 19:**
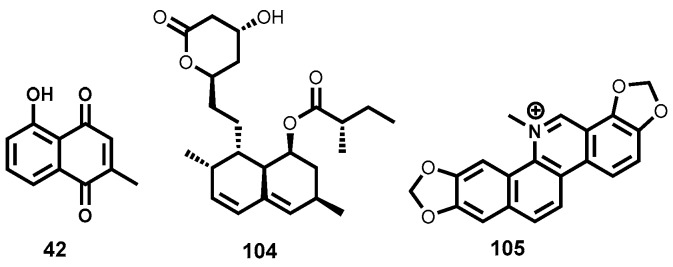
Structures of Mevinolin (lovastatin), plumbagin and sanguinarine.

Mevinolin (**104**) (Figure 19) is the template cholesterol lowering drug from which newer generation derivatives have been synthesised. The compound is produced by the fungus *Aspergillus terreus* and is an inhibitor of the enzyme 3-hydroxy-3-methylglutaryl-coenzyme A (HMG-CoA) reductase involved in the cholesterol synthesis pathway [248]. In an Antischistosomal assay, mevinolin was found to decrease the production of eggs, an action thought to arise from inhibition of the synthesis of dolichol-like compounds crucial to the modification (mannosylation) of proteins in the schistosomes, hence impairing their activities. Mevinolin, on prolonged exposure, displayed activity on both adult and juvenile worms [249,250].

When tested at 10 µM—the WHO recommended cut-off for an antischistosomal hit-plumbagin (**42**) and sanguinarine (**105**) (Figure 19) produced various morphological changes resulting in parasite death. In addition to the greater potency of sanguinarine over plumbagin against adult worms, the diversity of detrimental alterations produced suggest that the two compounds may have different modes of antischistosomal action [251].

#### 4.3.13. Summary

Despite the considerable public health effect of schistosomiasis, drug discovery efforts remain disproportionate. Natural products have been explored with the aim of identifying novel leads for this parasitic disease. Many of the tested agents derive their history from culinary and other medicinal uses while for others, only crude extracts have been investigated. Major efforts are still needed to identify isolated chemical compounds responsible for antischistosomal activity. Moreover, further assays to evaluate cytotoxicity and pharmacokinetics are requisite to be able to identify potential antischistosomal drug leads. The most advanced agents tested for potential use in the treatment of schistosomiasis hitherto are the quinine analogues and artemisinin-based antimalarial drugs, inspired mainly by their established use in the treatment of malaria. The successful clinical trials by these agents provide confidence in the use of natural products in this parasitic disease.

## 5. Lymphatic Filariasis

### 5.1. Background of the Disease

Lymphatic filariasis, commonly referred to as elephantiasis is a parasitic infection caused by the *Wuchereria bancrofti*, *Brugia malayi* and *Brugia timori* filarial worms [252]. The World Health Organization estimated in October 2016 that 947 million people living in 54 countries worldwide are at risk of contracting lymphatic filariasis with the highest burden being in African countries [253]. Current treatment involves the use of ivermectin, diethylcarbamaine and albendazole. Public health efforts to eliminate lymphatic filariasis have involved the use of mass drug administration of combinations of the three drugs. However, these drugs mainly target the microfilarial stages of the parasite and are not effective on the adult worm [254]. There is, therefore, a need to develop drugs for this neglected tropical disease.

### 5.2. Naturally Derived Compounds for Lymphatic Filariasis

#### 5.2.1. Plant Origin

**Figure 20:**
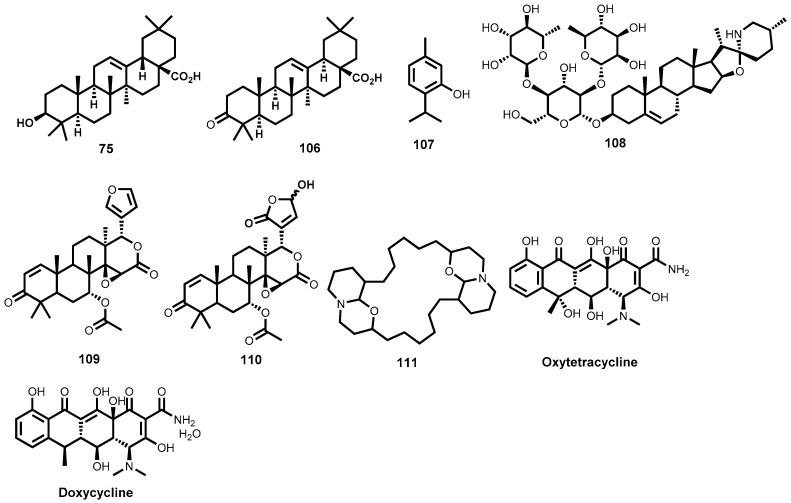
Naturally derived antifilarial compounds.

*Lantana camara*, a common plant that is native to tropical America and now regarded as a global weed was investigated for antifilarial activity. The crude extract administered in vivo to the *Mastomys coucha* rodent model at a high dose of 1 g/kg body weight for five days killed 43.05% of the adult *Brugia malayi*. Two compounds—oleanolic acid (**75**) and oleononic acid (**106**) (Figure 20)—isolated from *L. camara* exhibited antifilarial activity with an LC_100_ at 68.4 and 137 µM respectively [255].

The methanolic extract of *Trachyspermum ammi* fruits was found to possess activity against the adult bovine filarial worm, *Setaria digitata*. The crude extract had an IC_50_ of 0.067 mg/mL at 24 h and an IC_50_ of 0.019 mg/mL at 48 h. Higher potency was observed after a longer exposure period implying that this extract was slow acting [256]. Bioassay guided fractionation was carried out and thymol (**107**, Figure 20), a monoterpene phenolic compound was isolated. Like the crude extract, thymol was slow acting with an IC_50_ of 160 μM at 24 h and 13.3 μM at 48 h. The in vivo effect of thymol was tested against *Brugia malayi* in a *Mastomys coucha* model and the mean percentage of parasite mortality was 58.93% following a 50 mg/kg dose, while the control group had a mean percentage mortality of 19.05% [256].

A steroidal glycoalkaloid, solamargine (**108**, Figure 20), isolated from the ripe fruits of *Solanum khasianum* displayed in vitro activity against the adult worms and microfilaria of *Brugia malayi*, killing 100% at a concentration of 4 mg/mL in 60 min for the adult and 88 min for the microfilaria [252]. Total synthesis of solamargine and its analogues has been achieved thus it is possible to further explore this compound with the aim of improving activity and its drug like properties [257].

The crude aqueous and methanolic extracts of *Xylocarpus granatum* commonly referred to as the mangrove plant demonstrated in vitro antifilarial activity against the adult worm. Two compounds isolated from this plant displayed good antifilarial activity: Gedunin (**109**) IC_50_ value: 0.50 µM; photogedunin (**110**) IC_50_ 0.41 µM (Figure 20) [258].

Other drug discovery approaches that have been explored from plant sources include the synergistic effects of conventional and herbal therapy. Sharma et al., tested the effects of the methanolic extracts of *Vitex negundo* and *Aegle marmelos* when administered with diethylcarbamazine (DEC) and found that the extracts enhanced the microfilarial effects of DEC. However, attempts to fractionate the extracts led to loss of activity thus implying that the activity of the extracts may be due to synergism of the phytochemicals present as opposed to a single compound [259].

Another drug discovery approach that has been attempted is the target based drug discovery targeting the glutathione *S*-transferase (GST) enzyme found in filarial worms. Glutathione S transferase inhibitors obtained from plant sources such as ethacrynic acid, plumbagin and curcumin were found to inhibit the GST enzyme isolated from the female adult bovine filarial worm, *Setaria digitata* [260]

#### 5.2.2. Microbial Sources

The discovery of *Wolbachia*, an endobacterium found in filarial worms provided a promising target for chemotherapy by opening up the possibility of antibiotic therapy in the management of filariasis. Anti-rickettsial agents like tetracycline, doxycycline (Figure 20) and ciprofloxacin have been shown to deplete Wolbachia from the worms [261]. Doxycycline, a synthetic derivative of Terramycin, an antibiotic isolated from soil bacterium *Streptomyces rimosus* has demonstrated significant activity against macrofilaria. A daily dose of 200 mg was administered in a 3-, 4- and 6-week regimen. The 6-week regimen showed microfilaricidal activity of 92%, the 4-week regimen had 83% while no activity was observed in the 3-week regimen implying that there may be a minimum cumulative dose required for activity [262].

#### 5.2.3. Marine Sources

The marine sponge, *Haliclonia exigua* was investigated for antifilarial activity. The crude methanolic extract and the *n*-butanol soluble fractions killed the adult *Brugia malayi* worm at 31.25 µg/mL while the chloroform extract exhibited higher activity, killing the worms at 15.6 µg/mL. Araguspongin C (**111**, Figure 20) was isolated from this organism and found to have activity that was similar to that of the crude extract (IC_50_ = 34.9 µM). When tested in vivo, both the crude extract and Araguspongin C did not exhibit any significant activity [263]. The related species, *Haloclonia oculata* chloroform extract exhibited in vitro activity against the adult *Brugia malayi* with an IC_50_ value of 1.80 µg/mL. Unlike the *H. exigua* extract, the *H. oculata* extract exhibited significant in vivo microfilarial efficacy (64%) on the *B. malayi* intraperitoneal transplanted jird model [264].

### 5.3. Semi-Synthetic/Total Synthetic Compounds Based on Natural Compound Templates

#### Pre-Clinical Compounds

**Figure 21:**
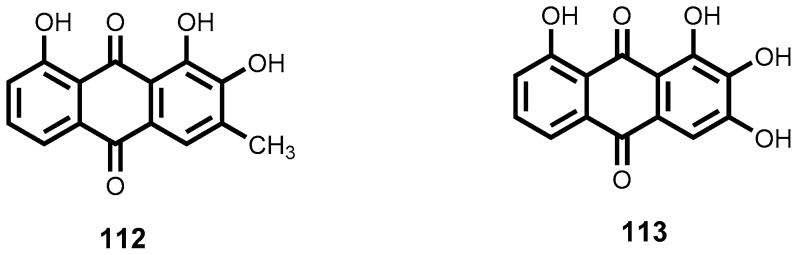
Synthetic antifilarial quinones based on natural product scaffolds.

A bioactive anthraquinone, anthraquinone J (**112**, Figure 21) isolated from daylily roots exhibited significant activity against *B. malayi* and was thus synthesized alongside a number of its analogues. Anthraquinones A–S were synthesized and tested against microfilaria and adult worms. Anthraquinone K (**113**, Figure 21) showed 100% mortality in 1 day against microfilaria; 3 days against female worms and 5 days against the adult male worms [265].

## 6. Concluding Remarks

NTDs remain a public health issue for emerging countries. As pointed out in the earlier sections of this review, their public health impact may well have been underestimated. Despite being ancient diseases, they have continued afflicting significant proportions of the world especially the world’s poorest. Investment, financial and otherwise, in the development of chemotherapeutic interventions against NTDs has been hampered, in part, by the general disregard of diseases afflicting poor populations as well as a prospect of limited financial returns on investment.

Current clinically used drugs against NTDs are beset by numerous shortfalls including toxicity, complicated administration procedures, limited availability and emergence of resistance. Natural products represent an untapped source of novel and structurally diverse chemotypes with potential to act via novel mechanisms of action against various causative agents of NTDs. In this review article we have reviewed natural products and their derivatives as potential leads for new therapies against NTDs. We have highlighted the immense therapeutic potential that remains untapped in natural product-derived compounds. Numerous metabolites isolated from plant, microbial and marine sources have been discussed. A limited number of synthetic and semisynthetic compounds motivated by natural product scaffolds have also been covered. Highly potent classes of metabolites from different chemical classes—alkaloids, phenolic compounds, quinones, terpenes, saponins, lignans, taxoids, anthranoids among others, have been discussed. Although a myriad of different classes of metabolites have demonstrated comparable and, in some cases, superior potency along with better toxicity profiles, compared to standard drugs, none of the promising leads have been progressed further into clinical development. There is need to seriously consider further clinical development of some promising natural products. The natural product compounds covered in this review could be clinical candidates in their own right or could provide templates for further medicinal chemistry optimization programmes.
